# Single sample resolution of rare microbial dark matter in a marine invertebrate metagenome

**DOI:** 10.1038/srep34362

**Published:** 2016-09-29

**Authors:** Ian J. Miller, Theodore R. Weyna, Stephen S. Fong, Grace E. Lim-Fong, Jason C. Kwan

**Affiliations:** 1 Division of Pharmaceutical Sciences, University of Wisconsin–Madison, Madison, Wisconsin 53705, U.S.A; 2 Department of Chemical and Life Science Engineering, Virginia Commonwealth University, Richmond, Virginia 23284, U.S.A; 3 Department of Biology, Randolph-Macon College, Ashland, Virginia 23005, U.S.A

## Abstract

Direct, untargeted sequencing of environmental samples (metagenomics) and *de novo* genome assembly enable the study of uncultured and phylogenetically divergent organisms. However, separating individual genomes from a mixed community has often relied on the differential-coverage analysis of multiple, deeply sequenced samples. In the metagenomic investigation of the marine bryozoan *Bugula neritina*, we uncovered seven bacterial genomes associated with a single *B. neritina* individual that appeared to be transient associates, two of which were unique to one individual and undetectable using certain “universal” 16S rRNA primers and probes. We recovered high quality genome assemblies for several rare instances of “microbial dark matter,” or phylogenetically divergent bacteria lacking genomes in reference databases, from a single tissue sample that was not subjected to any physical or chemical pre-treatment. One of these rare, divergent organisms has a small (593 kbp), poorly annotated genome with low GC content (20.9%) and a 16S rRNA gene with just 65% sequence similarity to the closest reference sequence. Our findings illustrate the importance of sampling strategy and *de novo* assembly of metagenomic reads to understand the extent and function of bacterial biodiversity.

Much of our modern understanding of microbial ecology, physiology and evolution depends on our ability to compare genomes. However, it has long been known^1^ that our genomic catalog and bioinformatic techniques remain biased towards studying cultured organisms^2^,^3^, which represent a minuscule fraction of true microbial diversity^4^. The remaining majority, which contribute important biogeochemical functions^5^,^6^, are uncultured. Some of these uncultured microbes are phylogenetically divergent, lack complete genomes in reference databases and are said to be microbial “dark matter”^3^,^7^,^8^. While the metabolic capabilities and biotechnological potential of these divergent bacteria is currently inaccessible through laboratory culture, they can be investigated using culture-independent sequencing (metagenomics)^8^,^9^.

Metagenomic studies often rely on PCR amplification of conserved bacterial phylogenetic markers, such as the 16S ribosomal RNA gene^10^. Although the inherent biases in such an approach have long been known^11^,^12^,^13^, it was recently demonstrated by the Banfield group that a previously unknown region of the tree of life, the “candidate phyla radiation” (CPR), largely consists of bacteria that are inaccessible to standard 16S rRNA amplification protocols^14^. This phenomenon is due to primer mismatches and widespread intervening sequences that lead to non-canonical amplicon lengths^14^. Shotgun or random metagenomic sequencing^14^,^15^,^16^,^17^,^18^,^19^ is less biased than amplicon sequencing and, along with single-cell genomic approaches^3^,^7^,^20^,^21^, has been used to characterize unknown regions of bacterial phylogeny. Newly sequenced microbial dark matter genomes are typically very divergent from known species, which leads to difficulties in functional annotation approaches that rely on sequence similarity^21^. Because there are no reference sequences available for microbial dark matter, sequence similarity and reference-guided assembly cannot be used for these divergent organisms. Therefore, metagenomic sequencing efforts to date have often relied on extensive chemical or physical pre-treatment^14^ of samples prior to sequencing, and comparison of relative microbial abundance between multiple datasets^17^,^18^ to enable genome binning from *de novo* metagenomic assemblies. Assembly and binning by differential coverage^17^, requires multiple samples that have very similar genomic composition of dark matter organisms. Rare, novel genomes can also be assembled if many closely related metagenomes are available^18^. However, these methods are inadequate for observing high-resolution fluctuations in microbial dark matter among a small number of mass-limited samples that have a low amount of species overlap. Thus, the functional study of microbial dark matter transiently associated with mass-limited, rare or small hosts, such as marine invertebrates, could be improved if high quality genomes were attainable from individual samples with approaches that rely solely on information obtained from single samples, such as nucleotide composition^22^,^23^,^24^.

Here, we took a *de novo* shotgun metagenomic assembly approach to investigate bacteria associated with the marine bryozoan *Bugula neritina*. This sessile, colonial filter-feeding animal consists of individual zooids specialized for feeding, substrate attachment or reproduction (Supplementary Fig. S1). Feeding zooids capture phytoplankton with a lophophore, and the digested material is distributed throughout the colony via funicular cords. *Candidatus* Endobugula sertula – a vertically transmitted^25^, uncultured bacterial symbiont of *B. neritina* – is found in the funicular cords and larvae, which are chemically defended by symbiont-produced small molecules called bryostatins^26^. Sections of the biosynthetic pathway for the bryostatins have been sequenced through clone library methods^26^, but the full genome sequence of *Ca*. E. sertula has not been determined.

Our original objective was to assemble the genome of *Ca*. E. sertula and recover missing pieces of the *bry* pathway. Using shotgun metagenomic sequencing and *de novo* assembly we were able to recover the genome of this symbiont, which will be the subject of a separate report^27^. Because *Ca*. E. sertula was previously described as an almost complete monoculture in *B. neritina* larvae^25^,^28^, we were surprised to find evidence of seven additional bacteria associated with the brood chambers and adjacent autozoids^29^ of one individual host. Some of these genomes were remarkably divergent from known sequences and, collectively, could represent founding members of one new phylum, one new class, one new family and one new genus, many of which are not detectable with certain “universal” bacterial primers. The presence of these divergent genomes was sporadic among *B. neritina* individuals, or entirely unique to the sequenced sample, indicating that the bryozoan experiences dynamic shifts in the composition of its microbial associates. The assembly of multiple complete or near-complete microbial dark matter genomes from a single bryozoan illustrates the potential importance of single-sample resolution in future metagenomic studies.

## Results and Discussion

### Investigation of the *Bugula neritina* metagenome

We sequenced DNA from two different samples of *B. neritina* tissue – mature larval brooding chambers (ovicells and their adjacent feeding zooids (Supplementary Fig. S1)) from a single adult colony, termed AB1_ovicells, and pooled free-swimming larvae released from a collection of adults, termed MHD_larvae (Supplementary Table S1). Raw, unfiltered reads were first assembled into contigs using SPAdes^30^. The resulting metagenomic assembly was complicated by the presence of the eukaryotic host genome (~135 Mbp) and many short contigs (N50 2.6 and 1.6 kbp for AB1_ovicells and MHD_larvae, respectively, Supplementary Table S1). Reconstruction of 16S rRNA gene sequences from AB1_ovicells sequence reads using EMIRGE^31^,^32^, however, revealed evidence of multiple bacteria in addition to *Ca*. E. sertula (Supplementary Table S2). These additional genomes were found in AB1_ovicells, but not MHD_larvae, meaning that we could not use differential coverage^17^ to assemble them from shotgun sequence, although both metagenomes appeared to contain *Ca*. E. sertula, allowing us to identify and separate shared contigs belonging to this symbiont.

In order to effectively deconvolute bacterial genomes from AB1_ovicells, we took a sequential approach to simplifying the metagenome (Supplementary Fig. S2). Contigs were first classified taxonomically based on BLASTP^33^ searches of their translated predicted open reading frames (ORFs) against the non-redundant (nr) NCBI database. ORF taxonomic classifications were generated with MEGAN^34^, and contig taxonomies were decided through majority vote of the constituent ORF classifications using a custom Perl script, to reduce the influence of horizontal gene transfer (see Materials and Methods, SI Appendix). An initial simplification of the metagenome was achieved by removing all contigs classified as Eukaryota or unclassified at the kingdom level. This simplification left a set of predominantly bacterial contigs that appeared to form distinct groupings when GC% was plotted against coverage (Supplementary Fig. S3). Serendipitously, this set of contigs seemed to consist of well-separated groups that were taxonomically distinct, that could represent individual bacterial genomes (Supplementary Fig. S3).

A second simplification step was achieved by identifying contigs shared between the AB1_ovicells and MHD_larvae metagenomes, which should include any host genome contigs misclassified as bacteria and contigs belonging to *Ca*. E. sertula. Contigs in the ovicells tissue (AB1_ovicells) that also had read coverage in the larval tissue (MHD_larvae) formed two clusters based on GC% and coverage (Supplementary Fig. S4). These clusters were automatically separated using normal mixture modeling, a technique where data are fit to models of a varying number of groups with discrete distribution patterns^35^. The validity of this cluster-separation procedure was assessed by examining the assigned taxonomy of ORFs within each group (Supplementary Fig. S5). One of the groups shared between AB1_ovicells and MHD_larvae (clusters 1–2) was predominantly classified as the γ-proteobacterium IMCC1989^36^, as well as related bacteria such as *Teredinibacter turnerae*^37^. This taxonomy was consistent with previously reported phylogenies inferred from the *Ca*. E. sertula 16S rRNA sequence^36^,^37^,^38^,^39^. The other contig group in the shared contig pool appeared to be of mixed taxonomy including a large number of ORFs classified as Eukaryota, which likely represent host contigs misclassified as bacteria.

We then turned our attention to the remaining bacterial contigs unique to the AB1_ovicells assembly. Contigs were identified that contained a set of bacterial single-copy marker genes previously established by Rinke *et al*.^3^ to assess genome assembly completeness and contamination. This subset of contigs was clustered automatically with normal mixture modeling^35^ to give several additional clusters (Supplementary Fig. S6). Contig groups derived from this method showed a low level of single copy marker redundancy (Table 1).

**Figure 1:**
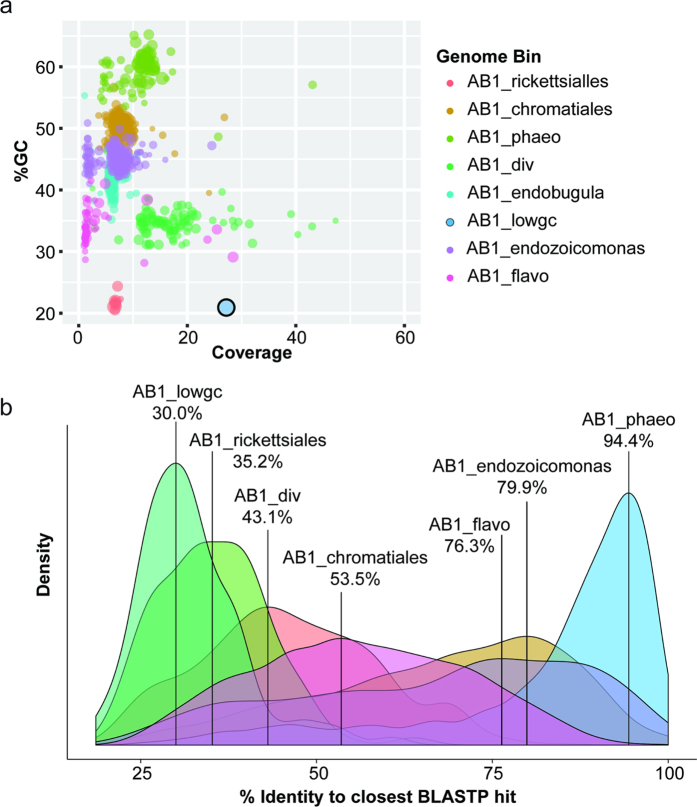
Separation and analysis of seven additional bacterial genomes isolated from the *Bugula neritina* AB1_ovicells metagenome. (**a**) %GC vs. coverage (*k*-mer coverage reported by the SPAdes assembler, where *k* = 77) plot of bacterial genome bins based on normal mixture modeling of conserved single copy gene marker-containing contigs. AB1_lowgc assembled into a single, circular contig and is outlined in black. (**b**) Density plot of highest % identity of predicted ORFs to known sequences in the respective genome assembly bins in BLASTP searches against NR. Bins are marked with the modal % identity. Divergent bins AB1_lowgc, AB1_rickettsiales and AB1_div show the lowest mode values.

The tetranucleotide frequencies of (clustered) contigs containing markers and other bacterial contigs were then analyzed with ESOM^22^, both to confirm the validity of automatically generated clusters, and to assign non-marker contigs to clusters with congruent tetranucleotide composition (Supplementary Fig. S7). Analysis of the resulting bacterial genome bins (Fig. 1 and Table 1), which were ultimately constructed using a combination of sequence homology and nucleotide composition, shows a varying level of estimated genome completeness by single-copy marker analysis^3^ and a low level of marker redundancy compared to what we could achieve with pre-established, fully automated binning algorithms, such as MaxBin^40^ (Supplementary Table S3). Analysis of the previously assigned ORF taxonomy in these respective bins showed that while four bins had fairly consistent taxonomic classifications (AB1_endozoicomonas, AB1_phaeo, AB1_flavo and AB1_chromatiales), three bins showed mixed ORF taxonomy even at the phylum level (AB1_lowgc, AB1_rickettsiales and AB1_div, Supplementary Fig. S8). The latter bins may represent highly divergent genomes that provide unreliable taxonomic classifications based on sequence similarity. Translated predicted ORFs in these bins were found to have very low identity to sequences within the nr database (the modal identities for AB1_lowgc, AB1_rickettsiales and AB1_div were 30.0%, 35.2% and 43.1%, respectively, Fig. 1b). AB1_lowgc was resolved to a single contig in the overall assembly, which we determined to be a circular chromosome by PCR and Sanger sequencing (Supplementary Table S4 and Fig. S9). Two divergent bins (AB1_lowgc and AB1_div) contained fully assembled 16S rRNA genes, which were found to not be amplifiable by certain universal bacterial primers, undetectable by eubacterial probe EUB338 and unassembled by EMIRGE, perhaps because of very low sequence similarity to reference sequences (Supplementary Table S5). These divergent bins, therefore, represent underexplored branches of the bacterial tree of life, or “microbial dark matter”^3^,^7^, that would be undetectable without shotgun metagenomic methods.

### Phylogenetic analysis of the divergent bacterial genomes in the *B. neritina* metagenome

**Figure 2:**
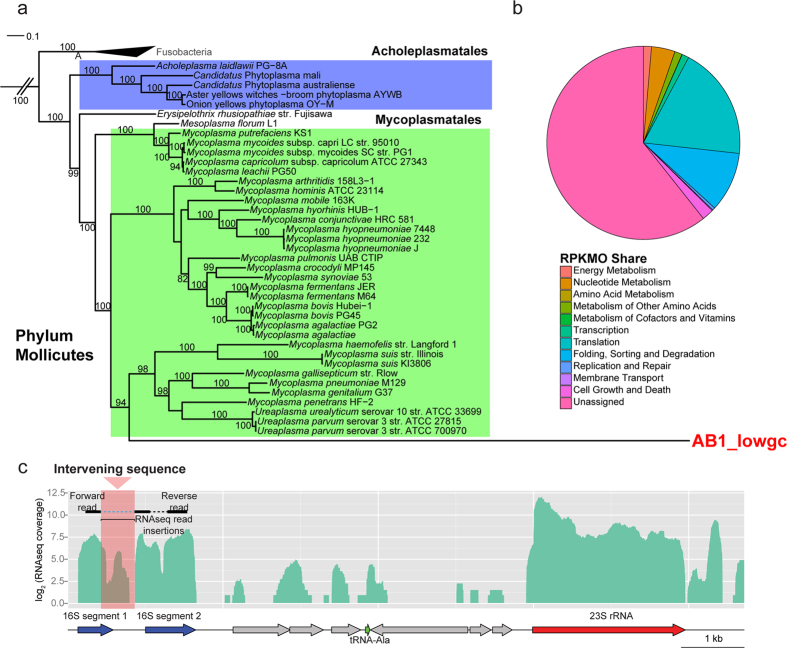
Phylogenetic and functional analysis of divergent AB1_lowgc genome. (**a**) Approximately maximum likelihood tree generated based on concatenated single-copy marker gene protein sequences from the AB1_lowgc genome assembly and 1,338 other reference genomes. Bootstrap proportions greater than 70% are expressed to the left of each node as a percentage of 1,000 replicates. (**b**) Proportion of normalized RNAseq reads, expressed as reads per kilobase of gene per million reads aligning to ORFs (RPKMO^53^) in the AB1_lowgc genome with assigned (39.3%) and unassigned (60.7%) function. (**c**) Plot of RNAseq read coverage against a locus in the AB1_lowgc chromosome containing 16S and 23S rRNA genes. Alignment to a structural model of the bacterial small ribosomal subunit (blue, Supplementary Fig. S23) shows two discrete homologous regions flanking an intervening sequence. This section appears as a gap in aligned RNAseq reads, indicating that it is excised from the mature RNA sequence. RNAseq coverage in the intervening sequence consists exclusively of reads that have no pairing partners in flanking genomic regions. The 23S rRNA gene is separated from the 16S rRNA gene, and the annotated region shown in red.

Where possible, taxonomies were assigned to each genome bin based on 16S rRNA gene sequence, using identity cutoffs recently suggested for high level taxa^10^ when comparing to the closest database sequence (Table 2). However, most genome bins did not contain assembled 16S rRNA genes, and so their phylogeny was initially investigated by constructing trees from alignments of concatenated marker protein sequences^3^ (Fig. 2a and Supplementary Figs S10–S15). These phylogenies suggested specific 16S rRNA gene sequences that had been directly reconstructed from paired reads using EMIRGE^31^,^32^ (Supplementary Table S2, Figs S16–S20), and it was possible for all bins except for AB1_flavo and AB1_chromatiales to join 16S rRNA sequences to contigs that contained fragments of an rRNA operon by PCR and Sanger sequencing (Supplementary Table S4).

Based on 16S rRNA sequence, AB1_div is most closely related to a known sequence (DQ395794), which is unclassified in the SILVA database beyond “Candidate division NPL-UPA2” (90%). In our 16S rRNA phylogenetic tree, this candidate division did not form a monophyletic group, but their sequences and the AB1_div sequence aligned with the Planctomycetes-Verrucomicrobia-Chlamydiae (PVC) superphylum, in agreement with the placement of AB1_div in the marker tree (Supplementary Fig. S12). However, while the 16S tree (Supplementary Fig. S16) placed AB1_div in a clade with Lentisphaerae and Chlamydiae, the protein marker tree placed AB1_div between Planctomycetes and Verrucomicrobia. Consistent with the placement of AB1_div in the PVC superphylum, we found a protein homologous to the PVC “Signature Protein” found by Lagkouvardos *et al*.^41^. As with many species in the PVC superphylum, we were not able to find the cell division protein FtsZ in AB1_div, but unlike many Planctomycetes and Chlamydiae species^42^, AB1_div contains the complement of genes enabling peptidoglycan synthesis (Supplementary Fig. S21). Analysis of other diagnostic insertions, deletions and signature proteins suggests that AB1_div has a common ancestor with all Planctomycetes, but represents a basal branch from this group (Supplementary Fig. S22).

Although an initial assembly of AB1_rickettsiales lacked a 16S rRNA gene, we noticed a low-coverage (2.4×), low-GC (28.6%) contig (“NODE_4002”) containing a full rRNA operon consistent with a divergent relative of α-proteobacteria in the order Rickettsiales, which was taxonomically congruent with the genome tree of AB1_rickettsiales (Supplementary Fig. S14). We used an iterative assembly algorithm in a custom Perl script (see Supplementary Information, Material and Methods), where reads are aligned to select contigs and re-assembled^43^, to improve the AB1_rickettsiales assembly and reveal where the contig “NODE_4002” joins previously assigned sequences (Table 1 and Supplementary Table S6). The joins suggested by this process were confirmed with PCR and Sanger sequencing. The original fragmented contig (“NODE_4002”) was 13.6 kbp in length, and was classified as order Rickettsiales based on ORF homology, but it was likely not grouped with the other AB1_rickettsiales contigs through ESOM because of high GC content skewed by the GC-rich rRNA operon on a relatively short contig (28.6% GC versus an average of 21.4% for assigned AB1_rickettsiales contigs).

AB1_rickettsiales appeared to be a divergent member of the order Rickettsiales in the genome tree (Supplementary Fig. S14); however, it has a contiguous rRNA region, in contrast to most of the Rickettsiales, in which the 16S rRNA gene is found in a different chromosomal region from the 23S and 5S rRNA genes^44^. This fragmentation is thought to have occurred in a common ancestor of the Rickettsiales, some time after the divergence of the mitochondrial line^44^. In a 16S rRNA-based tree, we found that AB1_rickettsiales forms a basal branch of the Rickettsiales, close to some members of “Rickettsiales genera incertae sedis” and the SAR11 clade (Supplementary Fig. S18). We found that, as in AB1_rickettsiales, the complete genomes available in the SAR11 group, as well as “incertae sedis” members *Ca*. Caedibacter acantamoebae (CP008936.1) and *Ca*. Paracaedibacter acanthamoaebae (CP008941.1) have contiguous 16S-23S-5S loci.

Rickettsiales are intracellular bacteria, either pathogens or symbionts capable of manipulating host cell reproduction^45^,^46^. They are common in insects and arachnids, which can act as vectors for other hosts^46^,^47^. These bacteria tend to have small, reduced genomes and display an interesting phenomenon of increased pathogenicity associated with decreased genome size and content^47^,^48^,^49^,^50^. Information from this genome bin suggests that it shares a number of features with currently available genomes in the order Rickettsiales. For instance, AB1_rickettsiales has a genome size of 436 kbp and the ADP/ATP carrier protein TlcA, a signature protein of intracellular bacteria enabling the leaching of host energy by the exchange of ADP for host ATP^51^,^52^, suggesting an intracellular and perhaps parasitic lifestyle for this organism. In common with the genomes of *R. prowazekii, R. bellii* and *O. tsutsugamushi*, AB1_rickettsiales appears to be lacking genes encoding flagellar assembly and chemotaxis^51^.

### Genomic and transcriptomic analysis of AB1_lowgc

To gain a functional snapshot of the most divergent genome (AB1_lowgc), we extracted RNA from AB1_ovicells and conducted RNA sequencing after depletion of ribosomal and polyadenylated (mostly eukaryotic transcript) RNA. Reads were aligned to our annotated genome assembly, and we calculated “RPKMO” (reads per kilobase of gene per million reads aligning to annotated ORFs in a given genome bin)^53^, which normalizes for both transcript length and coverage level, while eliminating the influence of rRNA coverage. Functional categories were assigned to predicted proteins using MEGAN^34^, and in order to assess the functions represented in each genome bin’s transcriptome, we assigned an “RPKMO share” (Fig. 2b) to each category by calculating the total RPKMO of all genes in that category as a proportion of total RPKMO for all ORFs.

AB1_lowgc is the bin most divergent from known taxa, with a 16S rRNA gene showing just 65% identity to *Ca*. Phytoplasma americanum (Phylum Tenericutes), which is below the phylum identity cutoff (75.0%) suggested by Yarza *et al*.^10^, and unassembled by the reference-guided program, EMIRGE^31^. AB1_lowgc has an unusual rRNA operon structure that was poorly annotated with automatic pipelines. Structural alignment of the 16S rRNA region with a rRNA model revealed an unaligned central 500 bp sequence inserted into the V4 region^10^ (Supplementary Fig. S23), which does not have homology with any sequence in the NCBI database. This is an example of an “intervening sequence” in the 16S rRNA gene, which has been observed in various Archaea^54^,^55^ and Bacteria^56^,^57^,^58^,^59^. A recent study has found that insertions in ribosomal RNA genes may be common in previously undetected bacteria in the “candidate phyla radiation” (CPR)^14^. We found evidence that this insertion is an intronic region, as alignments of RNAseq reads were consistent with the excision of the intervening sequence from the mature rRNA transcript (Fig. 2c).

Although the phylum of AB1_lowgc is uncertain, its genome shares features with its closest relatives in the Tenericutes phylum, the Mycoplasmas and *Candidatus* Phytoplasma spp., such as small size (593 kbp) and low GC content (20.9%). The Tenericutes phylum has an average GC content of 29.3% versus a bacterial average of 50.8%^60^. We did not find evidence of codon reassignment (Supplementary Table S7), and therefore AB1_lowgc may be more closely related to the Phytoplasmas (which use the normal bacterial genetic code) than the Mycoplasmas (which have reassigned the UGA stop codon to tryptophan)^61^. Further consistent with Phytoplasma^62^, we did not find many components of amino acid, nucleotide and fatty acid biosynthetic pathways (Supplementary Fig. S21), and perhaps more characteristically^63^,^64^, basic phosphotransferases associated with sugar import also appeared to be missing. This finding could suggest a similar level of genome reduction in AB1_lowgc (compared to *Candidatus* Phytoplasma spp.). However, as only ~20% of its protein-coding genes have any homologs in NCBI (Supplementary Fig. S9), we cannot determine the functions of the cryptic 80% of genes and therefore from comparison to available genomes it is difficult to judge the true extent of genome reduction. Only 129 out of 610 predicted protein-coding genes (21.1%) are functionally annotated (Supplementary Table S8 and Fig. S9), a much lower portion than some other founding representatives of candidate phyla considered to be microbial dark matter. By comparison, 56% of ORFs were functionally annotated when the first genome of the SR1 clade was sequenced^65^ and 43.1% of ORFs were functionally annotated for the first sequenced genome of the candidate phylum TM6^21^. Intriguingly, despite a general lack of annotated function, many of the “hypothetical” genes were highly expressed. For instance, seven of the top ten most highly expressed proteins by RPKMO in the AB1_lowgc transcriptome were unannotated beyond “hypothetical proteins” and 60.7% of RNAseq reads aligned to ORFs in the AB1_lowGC with unassigned function (Fig. 2b).

Among the genes that were functionally annotated, those related to translation as well as protein folding, sorting and degradation were highly expressed (Fig. 2b). This finding is consistent with previous studies of intracellular insect symbionts that have undergone significant genome reduction associated with an obligate intracellular lifestyle. In these systems, it is believed that folding and chaperone proteins are highly expressed in order to compensate for deleterious mutations accumulated through repeated replication bottlenecks^66^,^67^. One hallmark of genome reduction in obligate symbiosis is the loss of general transcriptional regulation^68^. However, differential levels of other annotated fractions, such as nucleotide metabolism (10.0%) cell growth and death (4.95%) and energy metabolism (3.45%), perhaps indicate that AB1_lowgc still maintains some element of transcriptional regulation. Yet based on limited functional annotation of its metabolic potential, AB1_lowgc seems to be lacking most mainstream metabolic pathways, including glycolysis and the tricarboxylic acid cycle. Its expressed metabolic functions are limited to ATP synthase, ribonucleotide reductase and nucleoside diphosphate kinase activities. Despite being highly expressed, only 3 components of an F_o_F_1_-type ATP synthase were functionally annotated, apparently suggesting that AB1_lowGC is missing portions of the complex in both the F_o_ and F_1_ domain or that the protein sequence for these domains is highly divergent from those in reference databases. We were unable to identify any signs of the subunit a or b of the F_0_, as well the *ε*-, ***γ***-, and *α*-subunits of the F_1_ domain^69^. AB1_lowgc’s sole annotated means of electron transport is limited to a thioredoxin reductase and in conjunction with some components of ATP synthase, it may be capable of energy generation via oxidative phosphorylation. However, it appears to lack any genes associated with fermentative metabolism and storage of complex carbon forms (e.g., polysaccharides).

### Dynamics of the *B. neritina* metagenome

**Figure 3:**
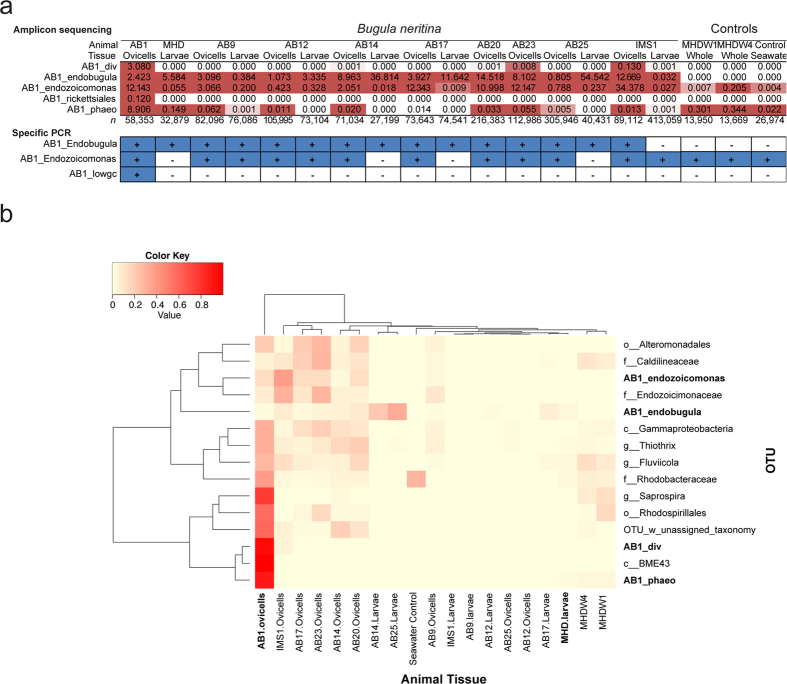
Spatial distribution and dynamics of AB1_ovicells bacteria. (**a**) Distribution of select bacteria (AB1_flavo and AB1_chromatiales genome bins lacked 16S rRNA genes) detected in AB1_ovicells in other *B. neritina* individuals, *B. stolonifera* (MHDW1 and MHDW4) and seawater by amplicon sequencing and specific PCR analysis. (**b**) Heat map of abundant (>1% relative abundance) AB1_ovicells OTUs displaying distribution, coincidence and relative abundance in other bryozoan tissue samples based on 16S rRNA amplicon sequencing.

Among the genome bins we detected in AB1_ovicells, only *Ca*. E. sertula has previously been described as a stable symbiont of *B. neritina*. We therefore sought to determine whether the other bacterial species we detected were found in other individual bryozoans. We subjected DNA extracted from additional reproductive animals to 16S rRNA amplicon sequencing (Fig. 3), and also carried out PCR studies with specific primers, since the 16S rRNA sequence of AB1_lowgc is not detectable with the amplicon primers used for high-throughput sequencing (Supplementary Table S5). Using a species cutoff of 98.7% identity^10^, amplicon detection of both AB1_endobugula and AB1_endozoicomonas largely agreed with PCR detection, except that specific primers did not detect AB1_endozoicomonas in some larval samples (Fig. 3a). This result may reflect an imperfect specificity of the ~430 bp amplicon used for high throughput sequencing, leading to potential false-positive detection in the amplicon data where closely-related species are present.

AB1_endozoicomonas was either not present or at low levels in larvae, but appeared to have a wide distribution in *B. neritina* adults. It also was potentially present at low levels in a co-occurring bryozoan (*B. stolonifera*) and seawater, perhaps indicating that it is a pervasive, free-living local strain that frequently associates with multiple bryozoan species. AB1_phaeo was sporadically detected in *B. neritina* and *B. stolonifera* and there was some evidence of AB1_div in multiple *B. neritina* samples; however, both of these are found at the highest levels in AB1_ovicells. By contrast, both AB1_rickettsiales and AB1_lowgc, which required screening based on custom primers, were found only in AB1_ovicells.

The AB1_ovicells metagenome, at least in terms of its most abundant OTUs detected by amplicon primers (>1% relative abundance), was distinct in its composition compared to all of the other samples we subjected to 16S rRNA amplicon sequencing (Fig. 3b). All abundant AB1_ovicells OTUs for which we constructed high quality genomes – except for AB1_flavo and AB1_chromatiales, as their genome assemblies lacked a 16S rRNA gene – were not detectable or were only minor components of other bryozoan samples. In fact, 44.2% of the amplicon reads representing abundant bacteria in AB1_ovicells were present in only that sample and by the same measure 91.3% of reads were found only in MHD_larvae (Supplementary Fig. S25). These results further suggest that the majority of bacterial associates in our two deeply sequenced datasets (AB1_ovicells and MHD_larvae) are only found in one bryozoan sample.

As a whole our data suggest that the population of bacteria associated with *B. neritina* is spatially dynamic, as the majority of bacteria in AB1_ovicells apart from *Ca*. E. sertula were not conserved among a sample of *B. neritina* individuals collected at the same location. This shift in composition would complicate efforts of bacterial contig binning based on differential coverage analysis, because the most divergent bacteria in AB1_ovicells were simply not present in all of the other samples. These genomes were also impossible to separate on the basis of sequence homology because of their extreme divergence from known sequences. The functional consequences of such transient associations with *B. neritina*, however, are as yet unknown.

## Summary

The larvae of marine bryozoan *B. neritina* were previously described as harboring a bacterial monoculture^25^,^28^ and the presence of other bacterial associates had not previously been investigated in adult animals. We found that several other bacterial genomes were major components of the AB1_ovicells metagenome, and were present in other animals to a varying degree. For instance, while AB1_endozoicomonas was pervasive in the samples examined, AB1_rickettsiales and AB1_lowgc were unique to AB1_ovicells.

Because of the predominantly non-overlapping composition of their bacterial associates, the two *B. neritina* shotgun metagenomes in this study were not amenable to deconvolution through differential coverage analysis^17^,^18^, a method devised for the reference-free assembly of multiple genomes from metagenomes. This approach would not have been able to deconvolute the multiple genomes unique to the AB1_ovicells metagenome, which included several novel bacteria we only observed once. Instead, we were successful with a hybrid approach using both nucleotide composition^22^,^23^,^24^ and homology-based simplification. It must be noted, however, that there are some limitations to our approach. As homology is used for the first round of simplification (separating bacterial from eukaryotic and unclassified contigs), it is conceivable that highly divergent microbes could be erroneously included in the “unclassified” bin. Only ~20% of genes in AB1_lowgc had any blast hits, and of those 92% were classified as Bacteria. As this genome contained long stretches of unclassifiable genes, we were only successful in capturing it because the chromosome was assembled in a single contig. A lower quality assembly would potentially lead to loss of some sections from the bin. Likewise, we are fundamentally limited by sequence coverage. For example, we reconstructed nine bacterial 16S rRNA genes with EMIRGE, and only recovered eight genomes, of which two contained 16S rRNA genes too divergent for EMIRGE to reconstruct. The extra 16S rRNA sequences reconstructed from reads are likely to belong to genomes that were at too low abundance to be assembled but nevertheless carried multiple 16S rRNA copies.

Fortunately, in this case the component genomes in the AB1_ovicells metagenome were sufficiently distinct in coverage and GC content to allow separation by normal mixture modeling from a single sample that was not subjected to any physical or chemical pre-treatment, ultimately allowing us to assemble high quality genomes and capture a functional snapshot of several divergent and rare instances of “microbial dark matter.” This term has been used to describe regions of the bacterial tree of life for which 16S rRNA sequences have been determined, but little other information is available. This sentiment, however, does not acknowledge the presence of bacterial groups whose 16S rRNA sequences have never been detected. This distinction has important implications for biology, as although there may be over 1,000 bacterial phyla, the rate at which novel 16S rRNA sequences are discovered has been steadily decreasing despite an exponential increase in sequences being deposited to public databases, leading to a prediction that “the rate of detection of new genera and new species [of bacteria and archaea] may be close to zero by the end of 2015 and 2017, respectively”^10^. However, this decrease is likely due to inherent limitations of “universal” 16S rRNA primers, whose design is restricted by the corpus of known sequences. In support of this notion, based on a study of Colorado groundwater, Banfield and colleagues recently found that a large and previously unidentified portion of the bacterial tree of life had 16S rRNA genes not amplifiable with standard primers^14^.

Our results here further suggest that we cannot currently know the full extent of bacterial diversity, as in one single sample we were able to find three genomes containing 16S rRNA sequences not amplifiable by the commonly used primer set 27F/1492R or detectable by the eubacterial probe EUB338, and one of these sequences was also not amplifiable by a primer set recently designed for metagenomic sequencing (Supplementary Table S5). Based on the similarity of the 16S rRNA genes of our assembled genomes to known sequences, the genomes found in AB1_ovicells could, collectively, represent founding members of one new phylum, one new class, one new family and one new genus. The extent to which the bacterial tree of life is extended from this one sample illustrates the likely extent of biodiversity yet to be found, and the need for approaches that can assemble rare, divergent bacteria unique to single samples.

## Materials and Methods

### Collection of biological material and preservation

Adult individual *B. neritina* animals were collected by hand from the sides of floating docks at three sites in November 2013 and April 2014 near Morehead City, NC, and maintained in flow-through seawater tanks until processing. Samples are designated by collection site - AB (N 34° 42′ 24.527″ W 76° 44′ 18.286″, 13 individual *B. neritina* colonies), MHD (N 34° 43′ 8.879″ W 76° 42′ 49.838″, ~20 *B. neritina* colonies used for combined larval collection plus two individual *B. stolonifera* colonies) or IMS (N 34° 43′ 19.823″ W 76° 45′ 855″, two *B. neritina* colonies). “Ovicells” samples were prepared by dissecting ovicell-rich sections of *B. neritina* animals containing larvae prior to release, while “larvae” samples were prepared by housing individual or multiple *B. neritina* colonies in a glass vessel, which was exposed to early morning sunlight. Free-swimming larvae released under these conditions were collected by pipet and stored on ice to aid settling and removal of excess seawater. Dissected ovicells or larval pellets were submerged in RNAlater (Sigma), incubated at room temperature for ~3 hr then stored at −80 °C.

### Other Experiments

Additional methods, along with supporting tables, figures and protocols are available in the SI Appendix.

## Additional Information

**Accession codes**: Annotated draft and complete genomes, as well as corresponding shotgun metagenome reads for AB1_endozoicomonas, AB1_flavo, AB1_chromatiales, AB1_div, AB1_endobugula, AB1_lowgc, AB1_rickettsiales and AB1_phaeo are all accessible through NCBI bioproject PRJNA322176.

**How to cite this article**: Miller, I. J. *et al*. Single sample resolution of rare microbial dark matter in a marine invertebrate metagenome. *Sci. Rep*. **6**, 34362; doi: 10.1038/srep34362 (2016).

## Supplementary Material

Supplementary Information

## Figures and Tables

**Table 1:** AB1_ovicells bacterial genome assembly characteristics.

*Bin*	*Contigs*	*Size (Mbp)*	*N50 (kbp)*	*Longest contig (kpb)*	*Coverage*†	*GC%*	*Completeness (%)*	*Duplicated markers*
*AB1_flavo*	315	1.59	4.64	56.3	3.3X	33.8	47.5	2
*AB1_chromatiales*‡	207	7.44	6.93	320.0	2.4X	50.4	100	2
*AB1_div*	108	1.91	27.3	102	20.8X	34.7	96.4	2
*AB1_endozoicomonas*	272	4.05	20.8	84.5	7.0X	45.3	82	15
*AB1_lowgc*	1	0.593	593	593	27.2X	20.9	23.0	0
*AB1_rickettsiales*‡	8	0.436	83.8	177.5	2.3X	21.6	48.9	0
*AB1_phaeo*‡	106	4.67	124.1	559.5	2.9X	60.3	98.6	3

^†^The coverage quoted here is *k*-mer coverage reported by the SPAdes assembler, where *k* = 77.

^‡^Result achieved through iterative assembly of contigs after binning. The initial bin characteristics are shown in Supplementary Table S6.

**Table 2:** Inferred/proposed taxonomy of AB1_ovicells bacterial genome assemblies.

Bin	Taxonomy	Rationale
AB1_flavo	Family Flavobacteriaceae	Concatenated protein tree (Supplementary Fig. S10)
AB1_chromatiales	Order Chromatiales	Concatenated protein tree (Supplementary Fig. S11)
AB1_div	Candidate division NPL-UPA2	90% 16S identity to DQ395794
AB1_endozoicomonas	Genus Endozoicomonas	97% 16S identity to JX280191 (uncultured Endozoicomonas bacterium)
AB1_lowgc	Kingdom Bacteria	65% 16S identity to DQ174122 (Ca. Phytoplasma americanum)
AB1_rickettsiales	Order Rickettsiales	85% 16S identity to HM128617 (uncultured Rickettsiales bacterium)
AB1_phaeo	Family Rhodobacteraceae	92% 16S identity to DQ395663 (uncultured Sulfitobacter bacterium)

## References

[b1] Hugenholtz P., Goebel B. M. & Pace N. R. Impact of culture-independent studies on the emerging phylogenetic view of bacterial diversity. J. Bacteriol. 180, 4765–4774 (1998). 9733676 10.1128/jb.180.18.4765-4774.1998 PMC107498

[b2] Pop M. Genome assembly reborn: Recent computational challenges. Brief. Bioinform. 10, 354–366 (2009). 19482960 10.1093/bib/bbp026 PMC2691937

[b3] Rinke C. . Insights into the phylogeny and coding potential of microbial dark matter. Nature 499, 431–437 (2013). 23851394 10.1038/nature12352

[b4] Rappé M. S. & Giovannoni S. J. The uncultured microbial majority. Annu. Rev. Microbiol. 57, 369–394 (2003). 14527284 10.1146/annurev.micro.57.030502.090759

[b5] Falkowski P. G., Fenchel T. & Delong E. F. The microbial engines that drive Earth’s biogeochemical cycles. Science 320, 1034–1039 (2008). 18497287 10.1126/science.1153213

[b6] Wrighton K. C. . Fermentation, hydrogen, and sulfur metabolism in multiple uncultivated bacterial phyla. Science 337, 1661–1665 (2012). 23019650 10.1126/science.1224041

[b7] Marcy Y. . Dissecting biological ‘dark matter’ with single-cell genetic analysis of rare and uncultivated TM7 microbes from the human mouth. Proc. Natl. Acad. Sci. USA 104, 11889–11894 (2007). 17620602 10.1073/pnas.0704662104 PMC1924555

[b8] Lok C. Mining the microbial dark matter. Nature 522, 270–273 (2015). 26085253 10.1038/522270a

[b9] Handelsman J. Metagenomics: Application of genomics to uncultured microorganisms. Microbiol. Mol. Biol. Rev 68, 669–685 (2004). 15590779 10.1128/MMBR.68.4.669-685.2004 PMC539003

[b10] Yarza P. . Uniting the classification of cultured and uncultured bacteria and archaea using 16S rRNA gene sequences. Nat. Rev. Microbiol. 12, 635–645 (2014). 25118885 10.1038/nrmicro3330

[b11] Derakshani M., Lukow T. & Liesack W. Novel bacterial lineages at the (sub) division level as detected by signature nucleotide-targeted recovery of 16S rRNA genes from bulk soil and rice roots of flooded rice microcosms. Appl. Environ. Microbiol. 67, 623–631 (2001). 11157225 10.1128/AEM.67.2.623-631.2001 PMC92629

[b12] Paster B. J. . Phylogenetic analysis of the spirochetes. J. Bacteriol. 173, 6101–6109 (1991). 1917844 10.1128/jb.173.19.6101-6109.1991 PMC208357

[b13] Vergin K. L. . Screening of a fosmid library of marine environmental genomic DNA fragments reveals four clones related to members of the order Planctomycetales. Appl. Environ. Microbiol. 64, 3075–3078 (1998). 9687477 10.1128/aem.64.8.3075-3078.1998 PMC106819

[b14] Brown C. T. . Unusual biology across a group comprising more than 15% of domain Bacteria. Nature 523, 208–211 (2015). 26083755 10.1038/nature14486

[b15] Schofield M. M., Jain S., Porat D., Dick G. J. & Sherman D. H. Identification and analysis of the bacterial endosymbiont specialized for production of the chemotherapeutic natural product ET-743. Environ. Microbiol. 17, 3964–3975 (2015). 26013440 10.1111/1462-2920.12908 PMC4618771

[b16] Iverson V. . Untangling genomes from metagenomes: Revealing an uncultured class of marine Euryarchaeota. Science 335, 587–590 (2012). 22301318 10.1126/science.1212665

[b17] Albertsen M. . Genome sequences of rare, uncultured bacteria obtained by differential coverage binning of multiple metagenomes. Nat. Biotechnol. 31, 533–538 (2013). 23707974 10.1038/nbt.2579

[b18] Nielsen H. B. . Identification and assembly of genomes and genetic elements in complex metagenomic samples without using reference genomes. Nat. Biotechnol. 32, 822–828 (2014). 24997787 10.1038/nbt.2939

[b19] Mick E. & Sorek R. High-resolution metagenomics. Nat. Biotechnol. 32, 750–751 (2014). 25101744 10.1038/nbt.2962

[b20] Wilson M. C. . An environmental bacterial taxon with a large and distinct metabolic repertoire. Nature 506, 58–62 (2014). 24476823 10.1038/nature12959

[b21] McLean J. S. . Candidate phylum TM6 genome recovered from a hospital sink biofilm provides genomic insights into this uncultivated phylum. Proc. Natl. Acad. Sci. USA 110, E2390–E2399 (2013). 23754396 10.1073/pnas.1219809110 PMC3696752

[b22] Dick G. J. . Community-wide analysis of microbial genome sequence signatures. Genome Biol. 10, R85 (2009). 19698104 10.1186/gb-2009-10-8-r85 PMC2745766

[b23] Laczny C. C. . VizBin - an application for reference-independent visualization and human-augmented binning of metagenomic data. Microbiome 3, 1 (2015). 25621171 10.1186/s40168-014-0066-1 PMC4305225

[b24] Laczny C. C., Pinel N., Vlassis N. & Wilmes P. Alignment-free visualization of metagenomic data by nonlinear dimension reduction. Sci. Rep. 4, 4516 (2014). 24682077 10.1038/srep04516 PMC3970189

[b25] Sharp K. H., Davidson S. K. & Haygood M. G. Localization of ‘*Candidatus* Endobugula sertula’ and the bryostatins throughout the life cycle of the bryozoan *Bugula neritina*. ISME J. 1, 693–702 (2007). 18059493 10.1038/ismej.2007.78

[b26] Trindade-Silva A. E., Lim-Fong G. E., Sharp K. H. & Haygood M. G. Bryostatins: Biological context and biotechnological prospects. Curr. Opin. Biotechnol. 21, 834–842 (2010). 20971628 10.1016/j.copbio.2010.09.018 PMC4497553

[b27] Miller I. J., Vanee N., Fong S. S., Lim-Fong G. E. & Kwan J. C. Lack ofovert genome reduction in the bryostatin-producing bryozoan symbiont “Candidatus Endobugula sertula”. Appl Environ Microbiol 82, 000–000, doi: 10.1128/AEM.01800-16 (2016). 10.1128/AEM.01800-16 PMC5086551 27590822

[b28] Haygood M. G. & Davidson S. K. Small-subunit rRNA genes and *in situ* hybridization with oligonucleotides specific for the bacterial symbionts in the larvae of the bryozoan *Bugula neritina* and proposal of ‘*Candidatus* endobugula sertula’. Appl. Environ. Microbiol. 63, 4612–4616 (1997). 9361448 10.1128/aem.63.11.4612-4616.1997 PMC168781

[b29] Ostrovsky A. N. Evolution of Sexual Reproduction in Marine Invertebrates: Example of gymnolaemate bryozoans (Springer: Netherlands, , 2013).

[b30] Bankevich A. . SPAdes: A new genome assembly algorithm and its applications to single-cell sequencing. J. Comput. Biol. 19, 455–477 (2012). 22506599 10.1089/cmb.2012.0021 PMC3342519

[b31] Miller C. S., Baker B. J., Thomas B. C., Singer S. W. & Banfield J. F. EMIRGE: Reconstruction of full-length ribosomal genes from microbial community short read sequencing data. Genome Biol. 12, R44 (2011). 21595876 10.1186/gb-2011-12-5-r44 PMC3219967

[b32] Miller C. S. . Short-read assembly of full-length 16S amplicons reveals bacterial diversity in subsurface sediments. PLoS One 8, e56018 (2013). 23405248 10.1371/journal.pone.0056018 PMC3566076

[b33] Camacho C. . BLAST+: Architecture and applications. BMC Bioinformatics 10, 421 (2009). 20003500 10.1186/1471-2105-10-421 PMC2803857

[b34] Huson D. H. & Weber N. Microbial community analysis using MEGAN. Methods Enzymol. 531, 465–485 (2013). 24060133 10.1016/B978-0-12-407863-5.00021-6

[b35] Fraley C. & Raftery A. E. Model-based clustering, discriminant analysis, and density estimation. J. Am. Stat. Assoc. 97, 611–631 (2002).

[b36] Jang Y., Oh H.-M., Kim H., Kang I. & Cho J.-C. Genome sequence of strain IMCC1989, a novel member of the marine gammaproteobacteria. J. Bacteriol. 193, 3672–3673 (2011). 21602334 10.1128/JB.05202-11 PMC3133343

[b37] Yang J. C. . The complete genome of *Teredinibacter turnerae* T7901: An intracellular endosymbiont of marine wood-boring bivalves (shipworms). PLoS One 4, e6085 (2009). 19568419 10.1371/journal.pone.0006085 PMC2699552

[b38] Lim G. E. & Haygood M. G. ‘*Candidatus* Endobugula glebosa,’ a specific bacterial symbiont of the marine bryozoan *Bugula simplex*. Appl. Environ. Microbiol. 70, 4921–4929 (2004). 15294832 10.1128/AEM.70.8.4921-4929.2004 PMC492373

[b39] Lim-Fong G. E., Regali L. A. & Haygood M. G. Evolutionary relationships of ‘*Candidatus* endobugula’ bacterial symbionts and their *Bugula* bryozoan hosts. Appl. Environ. Microbiol. 74, 3605–3609 (2008). 18390670 10.1128/AEM.02798-07 PMC2423044

[b40] Wu Y.-W., Tang Y.-H., Tringe S. G., Simmons B. A. & Singer S. W. MaxBin: An automated binning method to recover individual genomes from metagenomes using an expectation-maximization algorithm. Microbiome 2, 26 (2014). 25136443 10.1186/2049-2618-2-26 PMC4129434

[b41] Lagkouvardos I., M.-A. J., Rattei T. & Horn M. Signature protein of the PVC superphylum. Appl. Environ. Microbiol. 80, 440–445 (2013). 24185849 10.1128/AEM.02655-13 PMC3911108

[b42] Fuerst J. A. Planctomycetes: Cell Structure, Origins and Biology (Humana Press, 2013).

[b43] Tsai I. J., Otto T. D. & Berriman M. Improving draft assemblies by iterative mapping and assembly of short reads to eliminate gaps. Genome Biol. 11, R41 (2010). 20388197 10.1186/gb-2010-11-4-r41 PMC2884544

[b44] Collins N. E. . The genome of the heartwater agent *Ehrlichia ruminantium* contains multiple tandem repeats of actively variable copy number. Proc. Natl. Acad. Sci. USA 102, 838–843 (2005). 15637156 10.1073/pnas.0406633102 PMC545511

[b45] Merhej V. & Raoult D. Rickettsial evolution in the light of comparative genomics. Biol. Rev. Camb. Philos. Soc. 86, 379–405 (2011). 20716256 10.1111/j.1469-185X.2010.00151.x

[b46] Werren J. H., Baldo L. & Clark M. E. Wolbachia: Master manipulators of invertebrate biology. Nat. Rev. Microbiol. 6, 741–751 (2008). 18794912 10.1038/nrmicro1969

[b47] Renvoisé A., Merhej V., Georgiades K. & Raoult D. Intracellular Rickettsiales: Insights into manipulators of eukaryotic cells. Trends Mol. Med. 17, 573–583 (2011). 21763202 10.1016/j.molmed.2011.05.009

[b48] Ogata H. . Mechanisms of evolution in *Rickettsia conorii* and *R. prowazekii*. Science 293, 2093–2098 (2001). 11557893 10.1126/science.1061471

[b49] Merhej V., Royer-Carenzi M., Pontarotti P. & Raoult D. Massive comparative genomic analysis reveals convergent evolution of specialized bacteria. Biol. Direct 4, 13 (2009). 19361336 10.1186/1745-6150-4-13 PMC2688493

[b50] Fournier P.-E. . Analysis of the *Rickettsia africae* genome reveals that virulence acquisition in *Rickettsia* species may be explained by genome reduction. BMC Genomics 10, 166 (2009). 19379498 10.1186/1471-2164-10-166 PMC2694212

[b51] Martijn J. . Single-cell genomics of a rare environmental alphaproteobacterium provides unique insights into Rickettsiaceae evolution. ISME J. 9, 2373–2385 (2015). 25848874 10.1038/ismej.2015.46 PMC4497978

[b52] Krause D. C., Winkler H. H. & Wood D. O. Cloning and expression of the *Rickettsia prowazekii* ADP/ATP translocator in *Escherichia coli*. Proc. Natl. Acad. Sci. USA 82, 3015–3019 (1985). 2986146 10.1073/pnas.82.9.3015 PMC397697

[b53] Mandlik A. . RNA-Seq-based monitoring of infection-linked changes in *Vibrio cholerae* gene expression. Cell Host Microbe 10, 165–174 (2011). 21843873 10.1016/j.chom.2011.07.007 PMC3166260

[b54] Burggraf S., Larsen N., Woese C. R. & Stetter K. O. An intron within the 16S ribosomal RNA gene of the archaeon *Pyrobaculum aerophilum*. Proc. Natl. Acad. Sci. USA 90, 2547–2550 (1993). 8460170 10.1073/pnas.90.6.2547 PMC46125

[b55] Itoh T., Suzuki K. & Nakase T. Occurrence of introns in the 16S rRNA genes of members of the genus *Thermoproteus*. Arch. Microbiol. 170, 155–161 (1998). 9683654 10.1007/s002030050628

[b56] Linton D. . Two types of 16S rRNA gene are found in *Campylobacter helveticus*: Analysis, applications and characterization of the intervening sequence found in some strains. Microbiology 140, 847–855 (1994). 7516795 10.1099/00221287-140-4-847

[b57] Linton D., Clewley J. P., Burnens A., Owen R. J. & Stanley J. An intervening sequence (IVS) in the 16S rRNA gene of the eubacterium *Helicobacter canis*. Nucl. Acids Res. 22, 1954–1958 (1994). 7518076 10.1093/nar/22.11.1954 PMC308106

[b58] Baker B. J., Hugenholtz P., Dawson S. C. & Banfield J. F. Extremely acidophilic protists from acid mine drainage host Rickettsiales-lineage endosymbionts that have intervening sequences in their 16S rRNA genes. Appl. Environ. Microbiol. 69, 5512–5518 (2003). 12957940 10.1128/AEM.69.9.5512-5518.2003 PMC194945

[b59] Salman V., Amann R., Shub D. A. & Schulz-Vogt H. N. Multiple self-splicing introns in the 16S rRNA genes of giant sulfur bacteria. Proc. Natl. Acad. Sci. USA 109, 4203–4208 (2012). 22371583 10.1073/pnas.1120192109 PMC3306719

[b60] Li X.-Q. & Du D. Variation, evolution, and correlation analysis of C+G content and genome or chromosome size in different kingdoms and phyla. PLoS One 9, e88339 (2014). 24551092 10.1371/journal.pone.0088339 PMC3923770

[b61] Sirand-Pugnet P., Citti C., Barré A. & Blanchard A. Evolution of mollicutes: Down a bumpy road with twists and turns. Res. Microbiol. 158, 754–766 (2007). 18023150 10.1016/j.resmic.2007.09.007

[b62] Thompson G. A. & van Bel A. J. E. Phloem: Molecular Cell Biology, Systemic Communication, Biotic Interactions (John Wiley & Sons, 2012).

[b63] Oshima K. . Reductive evolution suggested from the complete genome sequence of a plant-pathogenic phytoplasma. Nat. Genet. 36, 27–29 (2004). 14661021 10.1038/ng1277

[b64] Christensen N. M., Axelsen K. B., Nicolaisen M. & Schulz A. Phytoplasmas and their interactions with hosts. Trends Plant Sci. 10, 526–535 (2005). 16226054 10.1016/j.tplants.2005.09.008

[b65] Kantor R. S. . Small genomes and sparse metabolisms of sediment-associated bacteria from four candidate phyla. MBio 4, e00708–e00713 (2013). 24149512 10.1128/mBio.00708-13 PMC3812714

[b66] McCutcheon J. P. & Moran N. A. Extreme genome reduction in symbiotic bacteria. Nat. Rev. Microbiol. 10, 13–26 (2012). 22064560 10.1038/nrmicro2670

[b67] Fares M. A., Ruiz-González M. X., Moya A., Elena S. F. & Barrio E. Endosymbiotic bacteria: GroEL buffers against deleterious mutations. Nature 417, 398 (2002). 12024205 10.1038/417398a

[b68] Moran N. A., Dunbar H. E. & Wilcox J. L. Regulation of transcription in a reduced bacterial genome: nutrient-provisioning genes of the obligate symbiont *Buchnera aphidicola*. J. Bacteriol. 187, 4229–4237 (2005). 15937185 10.1128/JB.187.12.4229-4237.2005 PMC1151715

[b69] von Ballmoos C., Wiedenmann A. & Dimroth P. Essentials for ATP synthesis by F_1_F_0_ ATP synthases. Annu. Rev. Biochem. 78, 649–672 (2009). 19489730 10.1146/annurev.biochem.78.081307.104803

