# Supplementary material for: Single sample resolution of rare microbial dark matter in a marine invertebrate metagenome
Source: Sci Rep. 2016 Sep 29;6:34362. doi: 10.1038/srep34362 (PMC5041132; doi:10.1038/srep34362)
Supplement: Supplementary Information [file srep34362-s1.pdf]

Single sample resolution of rare microbial dark matter in a marine invertebrate metagenome

Ian J Miller[1], Theodore R Weyna[1], Stephen S Fong[2], Grace Lim-Fong[3] & Jason C Kwan[1]*

[1]*Pharmaceutical Sciences Division, University of Wisconsin-Madison, Madison, Wisconsin, USA.*
[2]*Department of Chemical and Life Science Engineering, Virginia Commonwealth University, Richmond, Virginia, USA.*
[3]*Department of Biology, Randolph-Macon College, Ashland, Virginia, USA.*

Supplementary Information

***Corresponding author**
email: jason.kwan@wisc.edu

SI Materials and Methods

DNA extraction and sequencing. Both *B. neritina* ovicells and larval samples preserved in RNAlater (Sigma) were subjected to a DNA extraction procedure previously optimized for tunicate microbiomes (1). Briefly, ovicells were ground with a mortar and pestle under liquid nitrogen, before being resuspended in 5 mL of 2 mg/mL lysozyme in TE. Larvae were added directly to 5 mL of 2 mg/mL lysozyme in TE. In both cases, extractions were incubated at 30 °C, with shaking, for 1 hr. After this time, 1.2 mL 0.5 M EDTA was added to each tube along with proteinase K (Qiagen, final concentration 0.2 mg/mL), and the mixtures were incubated at 30 °C for 5 min. After addition of 650 μL of 10% SDS, the mixtures were incubated at 37 °C with shaking overnight. NaCl (1.2 mL of 5M) was then added to each tube, along with 1.0 mL of CTAB/NaCl solution (10% CTAB in 0.7 M NaCl), and the tubes were incubated at 65 °C for 20 min. Mixtures were extracted twice with 1:1 phenol/chloroform, and 1 volume of isopropanol was added to the aqueous fraction, which was then stored at 4 °C overnight. Tubes were spun down at 3,220 *g* for 30 min at 0 °C. Supernatants were carefully removed and 2 mL 70% ethanol in water was added to each tube, before they were spun down again. Supernatants were removed and tubes were inverted for 20 min, before 500 μL of TE was added. The tubes were left overnight at 4 °C to allow DNA to dissolve, before extractions were subjected to repurification by Genomic Tip 100/G (Qiagen), according to the manufacturer's instructions. TruSeq (Illumina) libraries were prepared for both AB1_ovicells and MHD_larvae, with ~300 bp inserts. These were subjected to sequencing on an Illumina HiSeq 2000, in paired-end 101 bp runs. Sequence yields are shown in **Supplementary Table 1**.

RNA extraction and sequencing. Approximately 40 mg of AB1_ovicells tissue was ground with a mortar and pestle under liquid nitrogen, and then resuspended in 600 μL buffer RLT (Qiagen) containing 6 μL β-mercaptoethanol. The mixture was homogenized by drawing up and down a sterile 20G needle 15 times, before being spun down at 16,800 *g* for 3 min. Total RNA was then purified from the crude lysate using the RNeasy Mini kit (Qiagen), utilizing the optional DNase step. The resulting RNA was flash frozen in liquid nitrogen and stored at –80 °C. Prokaryotic and eukaryotic ribosomal RNA was depleted with the RiboZero rRNA removal (Epidemiology) kit (Epicentre), and eukaryotic polyadenylated transcripts were depleted with poly-T beads. RNA in the resulting eluate was recovered and purified with Agencourt RNA Clean XP beads. Stranded RNAseq Illumina libraries were

prepared with ~300 bp inserts, and subjected to two Illumina HiSeq 2000 sequencing runs, one paired-end 101 bp and one paired-end 151 bp. Sequence yields are shown in **Supplementary Table 1**.

Metagenomic assembly and deconvolution. Raw Illumina reads obtained from sequencing both AB1_ovicells and MHD_larvae were filtered with Seqyclean (2), using the parameters "-minimum read length 40 -qual 30 30". The resulting filtered reads were assembled with SPAdes 3.1.1 (3) using the parameters,"-k 33,55,77 --careful". Prodigal (4) was used to call and translate ORFs in contigs obtained from the AB1_ovicells dataset >3 kbp in length, using the "-m anon" parameter. The resulting translated sequences were used as queries in massively parallel BLASTP searches against the NR NCBI database, using a custom pipeline (5) designed to run parallel blast jobs on a distributed grid using HTCondor (6). Raw blast table outputs were processed with MEGAN (7) to yield NCBI taxonomy IDs for each ORF. These were used to assign taxonomy classifications to parent contigs based on majority vote of component ORF taxonomy IDs, prioritizing in descending order of taxonomic rank/specificity (i.e. species-level classifications were counted, if they were present, before more basal taxonomic levels were considered). Resulting taxonomy tables were used to visualize assemblies in R (8), using the ggplot2 package.

Quality filtered paired-end Illumina reads from the MHD_larvae dataset were aligned with Bowtie 2 (9) to all AB1_ovicells contigs >3 kbp that were classified as belonging to the kingdom Bacteria, using the "--very-sensitive" end-to-end read alignment option. The coverage of each contig in the resulting alignment was examined using the BedTools (10) component CoverageBed. Contigs with >1× MHD_larvae read coverage were separated and examined in R and found to comprise of two groups of contigs with different coverage and GC content (**Supplementary Figure 4**). These groups were separated in R with normal mixture modeling using the package mclust (11). One of these groups was found by single copy marker analysis (see below) to be a complete bacterial genome assembly identified as *Candidatus* Endobugula sertula due to the presence of *bry* pathway components and on taxonomic grounds. The other group contained a paucity of bacterial markers and likely consisted of mis-assigned host contigs.

The remaining AB1_ovicells contigs with <1× MHD_larvae read coverage were examined for additional bacterial genomes. Bacterial single copy marker genes were detected using HHMer3 (12) with an HMM database constructed from the set of PFAM accessions recently used by Rinke *et al.* to assess the genome completeness

of divergent single-cell genomes (13). HMM results were filtered using the cutoffs used by Rinke *et al.* (14) and contigs containing marker genes were annotated in the previously constructed taxonomy table. Contigs containing single copy markers were separated in R, and subjected to normal mixture modeling with the mclust package (11) to yield 11 clusters. Contig sets belonging to each cluster were separated and assessed for genome completeness (13). These cluster classifications were used to assist in the binning of other contigs by tetranucleotide frequency analysis (15) using ESOM (16) (**Supplementary Figure 7**). After tetranucleotide frequencies for all contigs were calculated and processed in ESOM, bins were constructed such that all marker-containing contigs were included, capturing other contigs with similar tetranucleotide frequencies. In some cases, clusters identified by normal mixture modeling did not form discrete groups on the ESOM-map. In these cases, outliers were excluded. The resulting genome assembly bins were subjected to an iterative assembly protocol, where the raw (unfiltered) HiSeq reads were realigned to contigs with Bowtie 2 (9) using the parameters ,"--end-to-end --very-sensitive --no-discordant --no-unal", followed by reassembly of all aligned reads and their pairs except for unpaired reads that aligned more than twice the library insert size from the ends of contigs. Only three genome bins (AB1_chromatiales, AB1_phaeo and AB1_rickettsiales) were significantly improved by this procedure (**Table 1, Main Paper, and Table S6).**

PCR amplification and screening to confirm connectivity between contigs. Primers (**Table S4**) were designed to have an annealing temperature of ~55 °C manually and using and the Primer3 algorithm (17) to test various aspects of genomic assemblies. For a 10 μL reaction matrix the following volumes and concentrations of each component were used: 5 μL 2× KOD Buffer, 2 μL 2 mM dNTPs, 0.2 μL KOD Xtreme Hot Start DNA Polymerase (Novagen), 1 μL 3 μM forward primer, 1 μL 3 μM reverse primer, and 0.8 μL template. Reactions were carried out on a Bio-Rad C1000 Touch Thermal Cycler in 8 vial 200 uL strip tubes using a thermocycle program consisting of 94 °C for 2 min, then 35 cycles of (98 °C for 10 s, 55 °C for 30 s, custom extension time [1 min per kbp expected product] at 68 °C), then 68 °C for 10 min with an indefinite hold at 12 °C upon thermocycle completion.

Bacterial genome annotation and RNAseq alignment. RNAseq sequencing data was filtered with Seqyclean, using the parameter "-polyat". The resulting filtered reads were aligned with Bowtie 2 (9) to contigs in each

bacterial genome bin using the end-to-end alignment options “--very-sensitive --no-discordant --no-unal”. To investigate the apparent intervening sequence in the AB1_lowgc 16S rRNA gene, the alignment was repeated with the gap-aware aligner Tophat2 (18) using the parameters “--b2-very-sensitive --library-type fr-firststrand”. The AB1_lowgc 16S rRNA region was also aligned to a structural model of the bacterial small ribosomal subunit using SSU-Align (19) (**Supplementary Figure 23**). For functional annotation, fasta files containing the sequences of contigs classified into their respective bins were annotated with the Prokka pipeline (20). For AB1_lowgc, the genbank file generated by Prokka was combined with RNAseq alignment files in Geneious (Biomatters Ltd.) to visualize transcript abundance. Reads aligned to each ORF were counted in Geneious, and for each gene normalized RPKMO (reads per kilobasepair of gene per million reads aligning to annotated ORFs in the AB1_lowgc genome) (21) values were calculated.

Construction of assembly phylogenetic trees. AMPHORA (22) was used to scan genome assemblies for phylogenetic marker genes, which were extracted and manually examined to resolve instances of multiple hits. The marker genes were individually aligned to the internal reference database supplied with AMPHORA. The set of individual marker alignments were filtered such that only reference genomes with >75% of the marker genes were retained, and then marker genes represented in <75% of these genomes were removed. The marker alignments were concatenated, and residues not aligning to AMPHORA’s HMM models, signified by lowercase residues in the resulting alignment file, were removed from the alignment. Trees were then constructed with FastTree 2 (23) using the parameters “-gamma -slow -spr 10 -mlacc 3 -bionj”. After FastTree 2 runs were complete, accession numbers were substituted for strain designations according to entries in the RefSeq database. All trees were rooted arbitrarily at the divergence of the phylum Deinococcus-Thermus and other bacteria, as others have done previously (22). Trees were manipulated using the Interactive Tree of Life server (24).

Construction of 16S rRNA phylogenetic trees. For AB1_phaeo, AB1_endozoicomonas and AB1_endobugula, 16S rRNA sequences for all type strains in the same order suggested by taxonomic classifications of contigs in the respective genome bin were downloaded from the Ribosomal Database Project (RDP) website (25), in aligned format. Because AB1_div, AB1_rickettsiales and AB1_lowgc showed inconsistent contig taxonomy classification

at the order level, comparison sequences were selected based on inferences from marker gene alignment trees (see above). These were downloaded from the SILVA database (26), as this database contains putative classifications for many sequences from uncultured sources in the PVC superphylum, candidate division NPL-UPA2 and the SAR11 clade. Sequence sets were uploaded to the RDP website for alignment, and the results were inspected manually in ClustalX and trimmed. FastTree 2 (23) was used to construct the trees, using the parameters "-slow -spr 5 -mlacc 3 -gamma -gtr -nt", and manipulated using the iTOL server (24).

Taxonomic assignment of genome assemblies. Taxonomic assignments (**Table 2, Main Paper**) were based on 16S rRNA sequence, where available. These sequences were used as queries in BLASTN searches against the SILVA database (26) to identify the closest relative. If the BLAST alignment encompassed the full length of the query sequence, the reported alignment identity was used for classification. Otherwise, the full sequence and its closest relative identified by BLAST were realigned in Geneious. Taxonomic assignments in relation to the SILVA or NCBI taxonomy of the closest relative were generated in accordance with the 16S identity thresholds suggested by *Yarza et al.* (27), species: 98.7%, genus: 94.5%, family: 86.5%, order: 82.0%, class: 78.5% and phylum: 75.0%.

Assessment of codon reassignment in AB1_lowgc. We used a procedure recently utilized in a study of stop codon reassignments in metagenomic sequences (28) to determine whether AB1_lowgc used genetic code 4 (as the mycoplasmas do) or genetic code 11 (as the phytoplasmas do). Briefly, we ran the AB1_lowgc chromosome sequence through the gene-finding program Prodigal (4) twice, first using genetic code 4 and then using genetic code 11. The ORFs assigned with both of these codes showed similar average lengths and cumulative scores (cscore, Supplementary Table 9), similar to *Ca*. Phytoplasma australiense. By contrast, the same procedure carried out on the genome of *Mycoplasma pneumoniae* showed a large reduction in both average ORF length and cscore when code 11 was used, versus code 4. Our results therefore do not show evidence of codon reassignment in the genome of AB1_lowgc.

Analysis of PVC superphylum signature proteins and indels in AB1_div. All instances of the PVC superphylum signature protein identified by Lagkouvardos *et al.* (29) were downloaded from the NCBI database,

and used as BLASTP queries against the predicted proteins of AB1_div. The top hit was used as a BLASTP query against NR, and the top hit in that search was found to be the PVC signature protein from *Candidatus* Kuenenia stuttgartiensis (Accession CAJ71823.1). Marker genes with characteristic insertions or deletions in the PVC superphylum as well as signature proteins for the phylum Chlamydiae identified by Gupta *et al.* (30, 31) were downloaded from the NCBI database and used as BLASTP queries against the predicted proteins of AB1_div. Putative hits were examined to determine if they were true homologs of the relevant gene, then aligned with the entire set in ClustalX (using the BLOSUM protein weight matrix), to determine if the AB1_div protein shared certain insertions or deletions.

Amplicon sequencing. A ~430 bp section of 16S rRNA genes was amplified from DNA extracts using primers S-D-Bact-0341-b-S-17 and S-D-Bact-0785-a-A-21 (38) (Supplementary Table 4) with additional custom 5´ ends specific to each sample. This custom section included MiSeq adapter sequences and a sample-identifying barcode sequence. Pooled amplicons were sequenced on an Illumina MiSeq instrument in a 2 × 250 bp paired-end run, expected to yield overlapping reads. Demultiplexed sequence read pairs were joined with Flash (32) and analyzed with QIIME (33). For downstream analysis, OTUs that did not have abundance >1 read in at least one sample were removed. Abundance of specific bacteria were measured by identifying OTUs that had 98.7% identity to the assembled or Sanger sequence.

Functional analysis of metatrascriptome data. Translated predicted protein sequences from the annotations of the AB1_lowgc genome bin were used as queries in BLASTP searches against the NR database (see **Metagenomic assembly and deconvolution**, above). The resulting BLAST result table was used as input to MEGAN (7), which was used to assign KEGG functional categories to protein sequences. KEGG trees were uncollapsed two levels in MEGAN, and all assignments except for “Organismal systems” and “Human diseases” were exported to a csv file (with the columns “Read name” and “KEGG name”). Pre-calculated RPKMO values and the MEGAN csv table were used to calculate proportions of the bin’s transcriptome that corresponded to each KEGG category. Where multiple KEGG categories were assigned to one predicted gene, that gene’s RPKMO value was split equally among the assigned categories.

1. Schmidt EW, Donia MS (2009) Chapter 23 Cyanobactin ribosomally synthesized peptides—A case of deep metagenome mining. *Methods in Enzymology* **458**:575–596.

2. Zhbannikov IY, Hunter SS, Settles ML (2013) SeqyClean User Manual, available at: https://github.com/ibest/seqyclean. [Accessed: June 22 2015].

3. Bankevich A, et al. (2012) SPAdes: A new genome assembly algorithm and its applications to single-cell sequencing. *J Comp Biol* **19**(5):455–477.

4. Hyatt D, LoCascio PF, Hauser LJ, Uberbacher EC (2012) Gene and translation initiation site prediction in metagenomic sequences. *Bioinf* **28**(17):2223–2230.

5. CHTC's BLAST Pipeline - Center for High Throughput Computing Available at: http://chtc.cs.wisc.edu/blast_SOARguide.shtml [Accessed June 22, 2015].

6. HTC Condor Available at: http://research.cs.wisc.edu/htcondor/index.html [Accessed June 22, 2015].

7. Huson DH, Weber N (2013) Microbial community analysis using MEGAN. *Methods in Enzymology.* **531**:465–485.

8. Gentleman R, Ihaka R, Bates D, Others (2009) The R project for statistical computing. *URL: http://www r-project org/254*. [Accessed: June 24 2015].

9. Langmead B, Salzberg SL (2012) Fast gapped-read alignment with Bowtie 2. *Nat Methods* **9**(4):357–359.

10. Quinlan AR, Hall IM (2010) BEDTools: A flexible suite of utilities for comparing genomic features. *Bioinf* **26**(6):841–842.

11. Fraley C, Raftery AE (2002) Model-based clustering, discriminant analysis, and density estimation. *J Am Stat Assoc* **97**(458):611–631.

12. Mistry J, Finn RD, Eddy SR, Bateman A, Punta M (2013) Challenges in homology search: HMMER3 and convergent evolution of coiled-coil regions. *Nucl Acids Res* **41**(12):e121.

13. Rinke C, et al. (2013) Insights into the phylogeny and coding potential of microbial dark matter. *Nature* **499**(7459):431–437.

14. Wilson MC, et al. (2014) An environmental bacterial taxon with a large and distinct metabolic repertoire. *Nature* **506**(7486):58–62.

15. Dick GJ, et al. (2009) Community-wide analysis of microbial genome sequence signatures. *Genome Biol* **10**(8):R85.

16. Ultsch A, Mörchen F (2005) ESOM-Maps: Tools for clustering, visualization, and classification with Emergent SOM. Technical report Available at: http://www.informatik.uni-marburg.de/~databionics/papers/ultsch05esom.pdf. [Accessed: June 22 2015].

17. Untergasser A, et al. (2012) Primer3—new capabilities and interfaces. *Nucl Acids Res* **40**(15):e115–e115.

18. Kim D, et al. (2013) TopHat2: Accurate alignment of transcriptomes in the presence of insertions, deletions and gene fusions. *Genome Biol* **14**(4):R36.

19. Nawrocki EP (2009) *Structural RNA homology search and alignment using covariance models*. Ph.D. thesis (Washington University in St. Louis).

20. Seemann T (2014) Prokka: Rapid prokaryotic genome annotation. *Bioinf* **30**(14):2068–2069.

21. Mandlik A, et al. (2011) RNA-Seq-based monitoring of infection-linked changes in *Vibrio cholerae* gene expression. *Cell Host Microb* **10**(2):165–174.

22. Wu M, Eisen JA (2008) A simple, fast, and accurate method of phylogenomic inference. *Genome Biol* **9**(10):R151.

23. Price MN, Dehal PS, Arkin AP, Others (2010) FastTree 2—approximately maximum-likelihood trees for large alignments. *PLoS One* **5**(3):e9490.

24. Letunic I, Bork P (2011) Interactive Tree Of Life v2: Online annotation and display of phylogenetic trees made easy. *Nucl Acids Res* **38**:W475–8.

25. Cole JR, et al. (2014) Ribosomal Database Project: Data and tools for high throughput rRNA analysis. *Nucl Acids Res* **42**(D1):D633–42.

26. Quast C, et al. (2013) The SILVA ribosomal RNA gene database project: Improved data processing and web-based tools. *Nucl Acids Res* **41**(D1):D590–596.

27. Yarza P, et al. (2014) Uniting the classification of cultured and uncultured bacteria and archaea using 16S rRNA gene sequences. *Nat Rev Microbiol* **12**(9):635–645.

28. Ivanova NN, et al. (2014) Stop codon reassignments in the wild. *Science* **344**(6186):909–913.

29. Lagkouvardos I, M.-A. J, Rattei T, Horn M (2013) Signature protein of the PVC superphylum. *Appl Environ Microbiol* **80**(2):440–445.

30. Gupta RS, Bhandari V, Naushad HS (2012) Molecular signatures for the PVC clade (Planctomycetes, Verrucomicrobia, Chlamydiae, and Lentisphaerae) of bacteria provide insights into their evolutionary relationships. *Front Microbiol* **3**:327.

31. Gupta RS, Griffiths E (2006) Chlamydiae-specific proteins and indels: Novel tools for studies. *Trends Microbiol* **14**(12):527–535.

32. Magoč T, Salzberg SL (2011) FLASH: Fast length adjustment of short reads to improve genome assemblies. *Bioinf* **27**(21):2957–2963.

33. Caporaso JG, et al. (2010) QIIME allows analysis of high-throughput community sequencing data. *Nat Methods* **7**(5):335–336.

34. Hyatt D, et al. (2010) Prodigal: Prokaryotic gene recognition and translation initiation site identification. *BMC Bioinf* **11**:119.

35. Miller CS, Baker BJ, Thomas BC, Singer SW, Banfield JF (2011) EMIRGE: Reconstruction of full-length ribosomal genes from microbial community short read sequencing data. *Genome Biol* **12**(5):R44.

36. Weisburg WG, Barns SM, Pelletier DA, Lane DJ (1991) 16S ribosomal DNA amplification for phylogenetic study. *J Bacteriol* **173**(2):697–703.

37. Reysenbach AL, Giver LJ, Wickham GS, Pace NR (1992) Differential amplification of rRNA genes by polymerase chain reaction. *Appl Environ Microbiol* **58**(10):3417–3418.

38. Klindworth A, et al. (2013) Evaluation of general 16S ribosomal RNA gene PCR primers for classical and next-generation sequencing-based diversity studies. *Nucl Acids Res* **41**(1):e1.

39. Amann RI, et al. (1990) Combination of 16S rRNA-targeted oligonucleotide probes with flow cytometry for analyzing mixed microbial populations. *Appl Environ Microbiol* **56**(6):1919–1925.

40. Haygood MG, Davidson SK (1997) Small-subunit rRNA genes and *in situ* hybridization with oligonucleotides specific for the bacterial symbionts in the larvae of the bryozoan *Bugula neritina* and proposal of "*Candidatus* endobugula sertula." *Appl Environ Microbiol* **63**(11):4612–4616.

41. Wu Y-W, Tang Y-H, Tringe SG, Simmons BA, Singer SW (2014) MaxBin: An automated binning method to recover individual genomes from metagenomes using an expectation-maximization algorithm. *Microbiome* **2**:26.

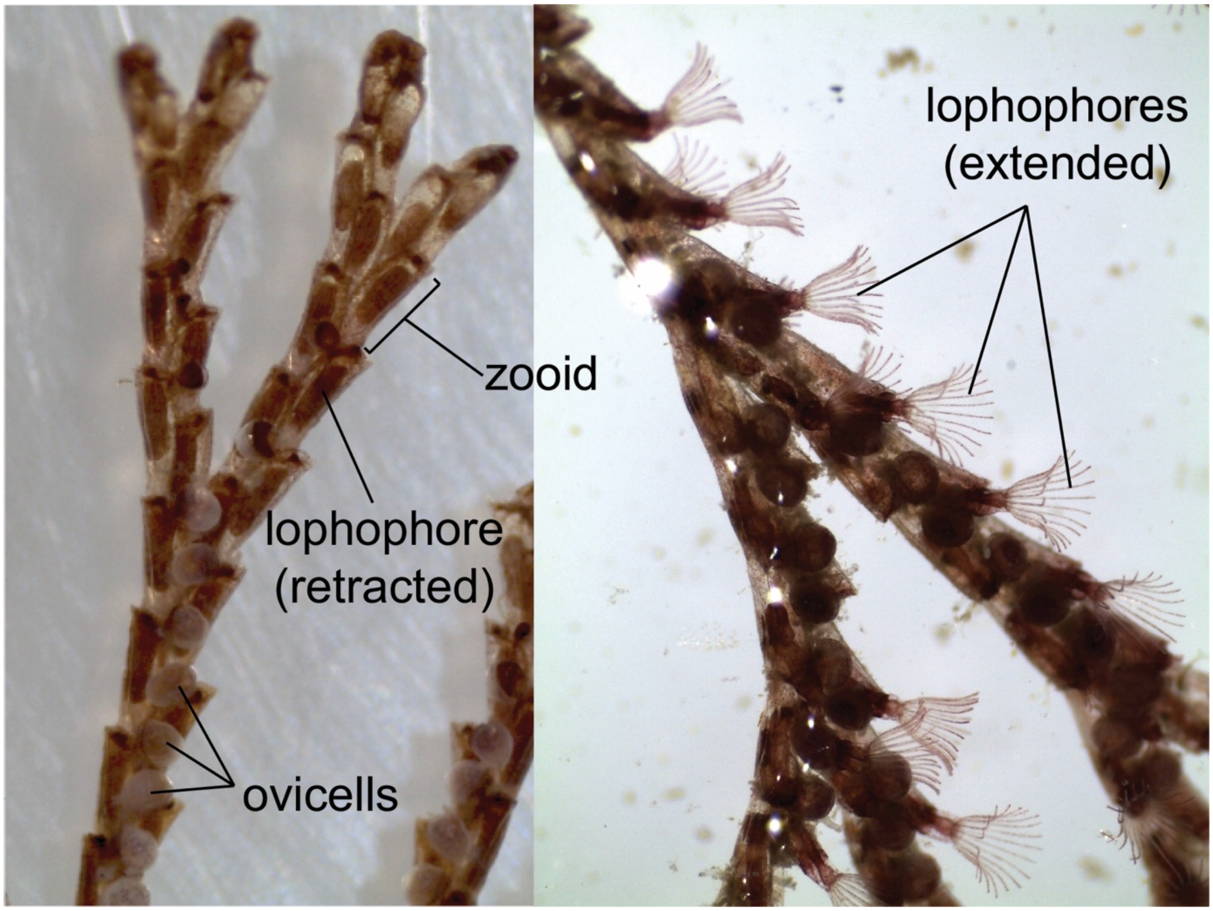

Supplementary Figure 1: Micrograph showing the morphology of zooids and ovicells in *B. neritina* colony. The colony consists of clonal zooids specialized for feeding, substrate attachment and reproduction. Feeding zooids capture suspended particles from the water through movement of their lophophore. Reproductive zooids hold a fertilized embryo inside an ovicell until the mature larva is released. The adult animal is covered in a protective layer of chitin, but the larvae are undefended except for chemical defenses (the bryostatins) produced by a vertically transmitted symbiont, *Candidatus* Endobugula sertula.

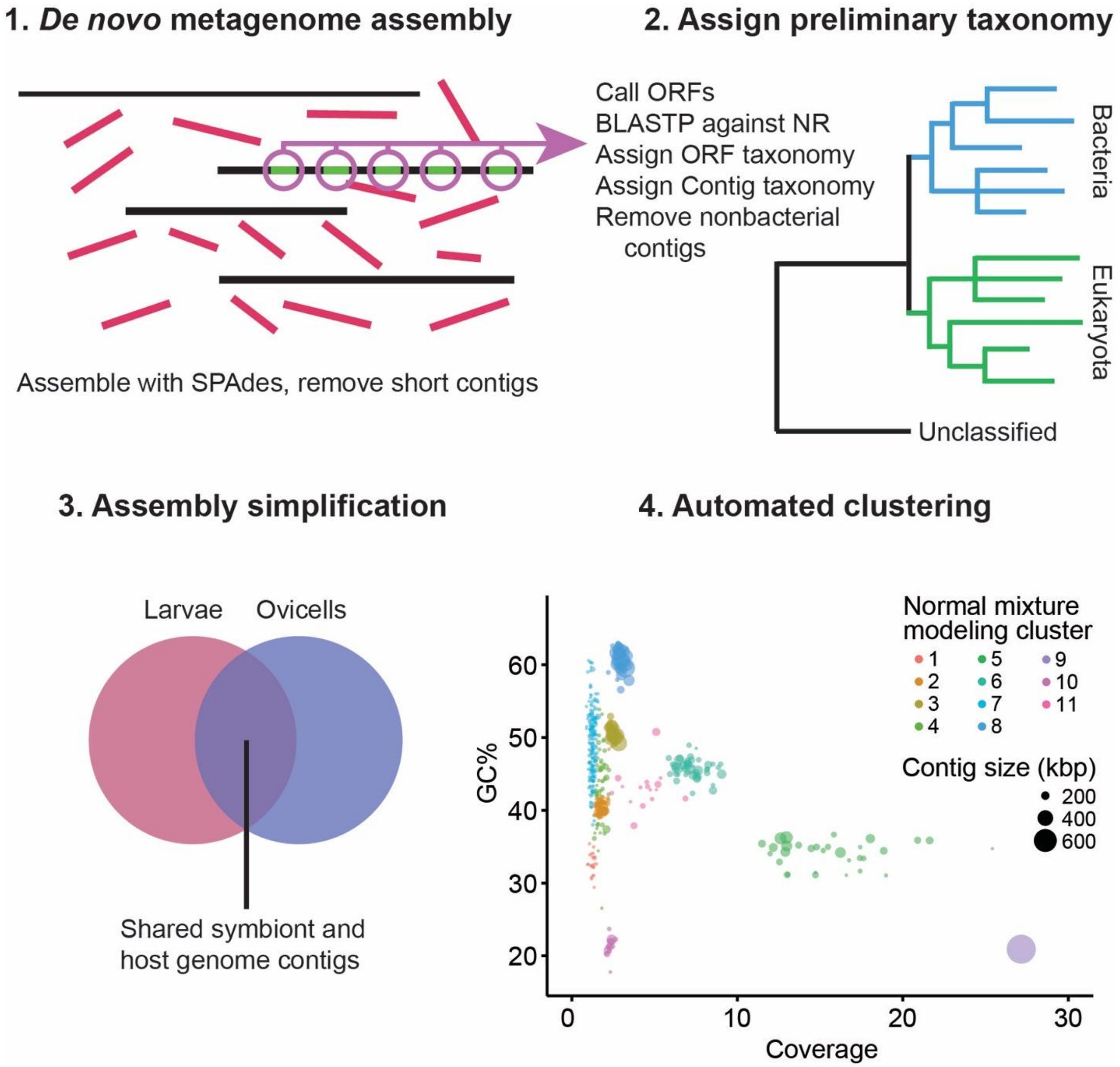

Supplementary Figure 2: Overview of metagenomic assembly and deconvolution process.
Overview of assembly and deconvolution process. **1.** Paired-end Illumina reads, obtained from AB1_ovicells DNA, were assembled, and contigs <3,000 bp were discarded. **2.** ORFs were called, translated, and used as queries in a BLASTP search against the NCBI database. The BLAST results were used to assign preliminary taxonomy to contigs. **3.** Paired-end Illumina reads, obtained from MHD_larvae DNA, were aligned to AB1_ovicells contigs classified in step 2 as belonging to the kingdom Bacteria, in order to identify conserved symbiont and host sequences. **4.** Bacterial contigs from AB1_ovicells that were not shared with MHD_larvae were searched for bacterial single-copy marker genes (13). Contigs containing markers were automatically clustered using normal mixture modeling (11). These clusters were used to assist binning of additional contigs based on tetranucleotide frequency.

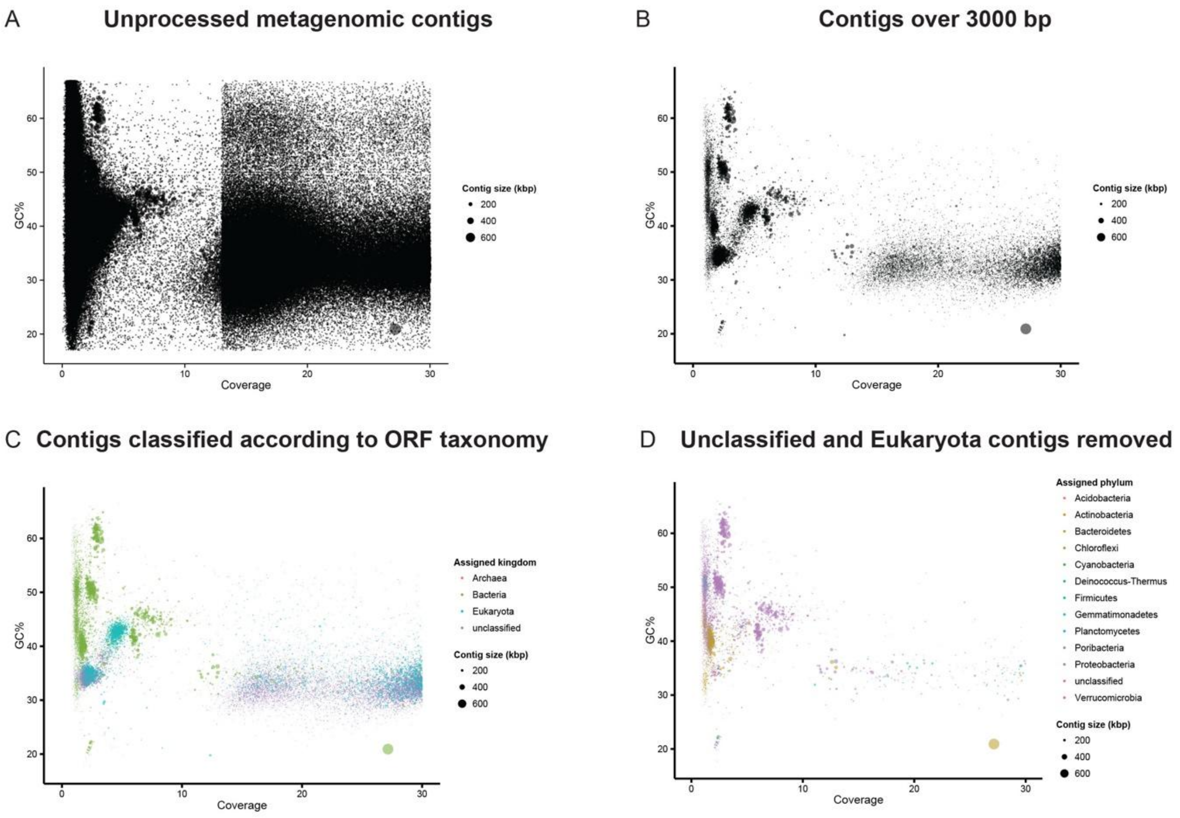

Supplementary Figure 3: Initial processing of AB1_ovicells metagenome. A. View of a portion of the unprocessed metagenome. **B**. In the first step, contigs with length <3,000 bp were removed. ORFs were called with Prodigal (34), and the translations were used as queries in BLASTP searches against the NCBI database. **C**. Contig taxonomies were assigned from the BLAST results with MEGAN (7) (see **Materials and Methods**). **D**. These taxonomy assignments were used to remove all contigs that were unclassified at the kingdom level or were classified as belonging to kingdom Eukaryota, which likely comprised of the host genome and other contaminating eukaryotic organisms.

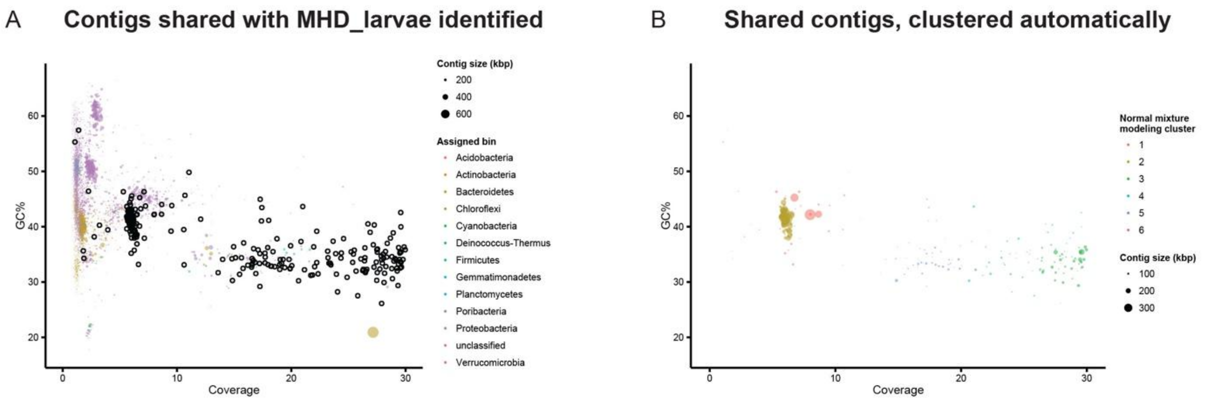

Supplementary Figure 4: Simplification of AB1_ovicells metagenome by comparison with MHD_larvae. Shotgun metagenomic sequence reads belonging to the MHD_larvae sample were aligned to bacterial contigs from the AB1_ovicells assembly. Contigs with >1× coverage with MHD_larvae reads were identified (**A**, circled in black) and separated. Distinct groups of contigs clustered according to coverage and GC% (**B**). These groups were assigned automatically using normal mixture modeling (11). Based on assigned contig and ORF taxonomy (Fig. S5), single-copy marker analysis, marker gene and 16S rRNA phylogeny, groups 1–2 were subsequently denoted AB1_endobugula (*Candidatus* Endobugula sertula). Groups 3–6 had mixed taxonomy (Fig. S5), including many ORFs classified as belonging to kingdom Eukaryota, and may represent contigs belonging to the host genome.

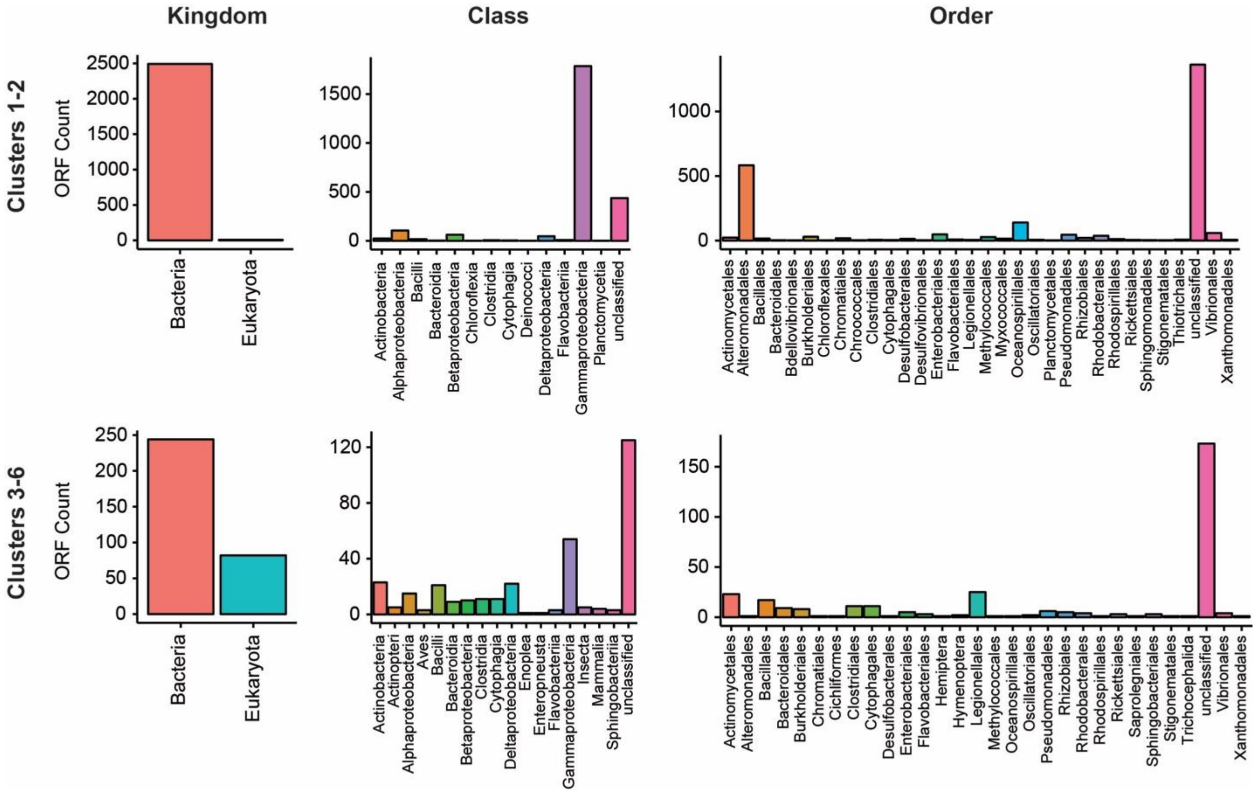

Supplementary Figure 5: Contigs shared between AB1_ovicells and MHD_larvae are automatically clustered into taxonomically distinct groups. Following normal mixture modeling (11) on the basis of coverage and GC% (**Fig. S4**), clusters 1 and 2 contain ORFs predominantly classified as gammaproteobacteria of the order Alteromonadales, consistent with the known phylogeny of *Ca.* E. sertula (see **top row**). By contrast, clusters 3–6 contain ORFs that are predominantly unclassified at phylogenetic levels below kingdom, with little consistency among classified ORFs (see **bottom row**).

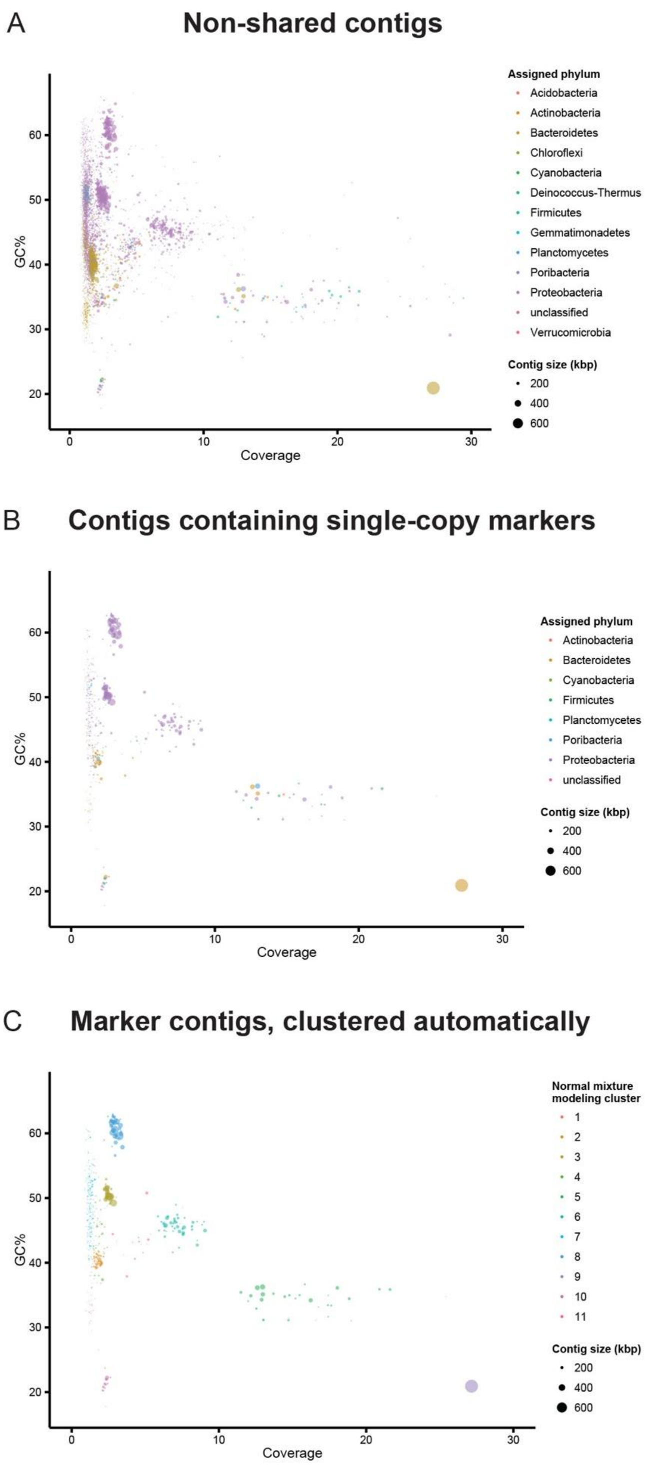

Supplementary Figure 6: Deconvolution of contigs unique to AB1_ovicells. Contigs unique to AB1_ovicells were separated (**A**), and bacterial single-copy marker genes were detected with the HMM models used in Rinke *et al.* (13). Contigs containing bacterial marker genes (**B**) were independently clustered using normal mixture modeling (11) (**C**).

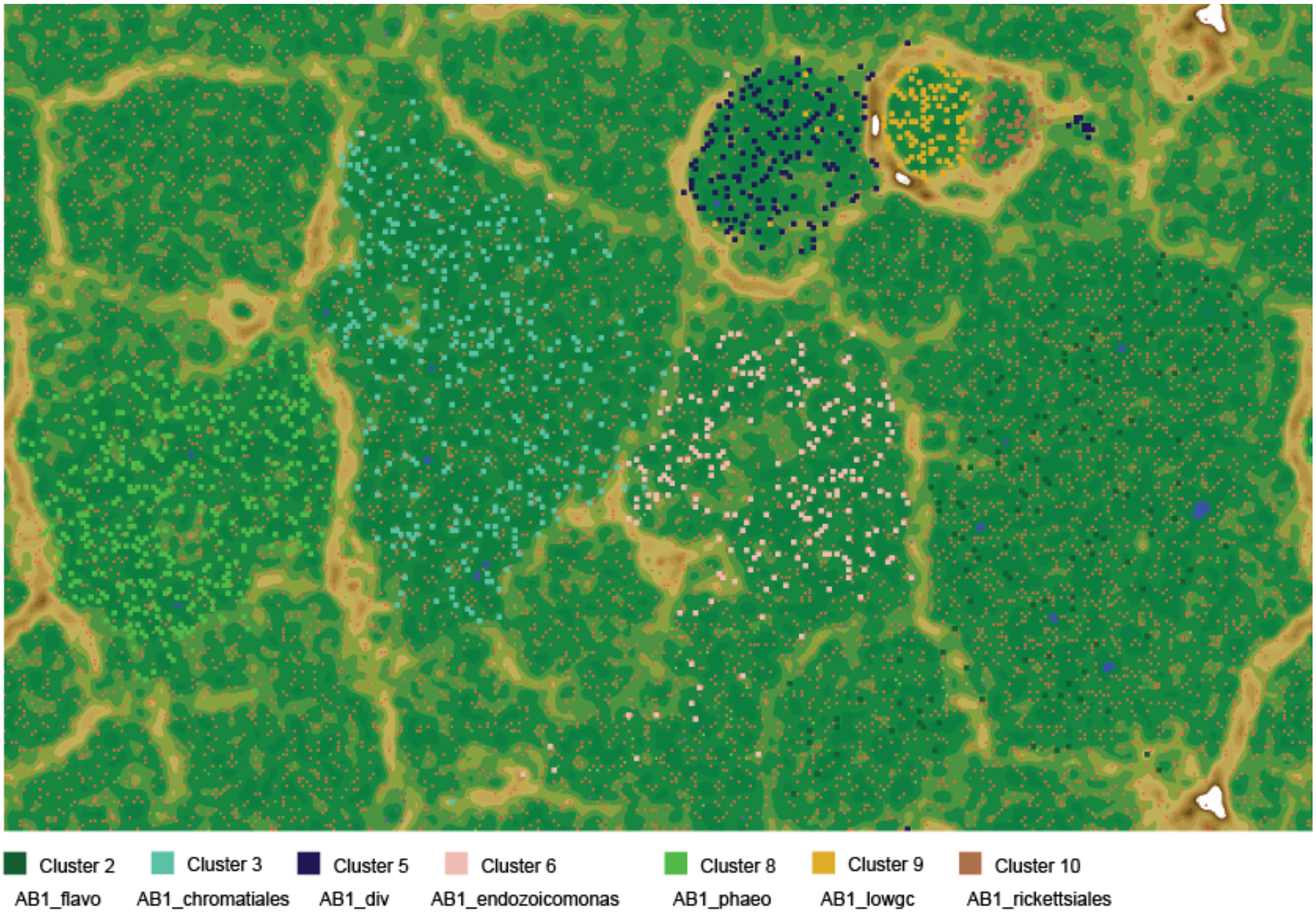

Supplementary Figure 7: AB1_ovicells contigs >3,000 bp in length, classified as bacterial but not found in MHD_larvae, were searched for bacterial single copy marker genes (see Materials and Methods**).** The subset of contigs containing single copy marker genes were clustered with normal mixture modeling (11) (**Fig. S2**). The ESOM-map (16) resulting from analysis of the complete set of contigs with and without marker genes is shown above. Points belonging to marker-containing genes are highlighted, with numbers corresponding to those shown in **Fig. 1, Main Paper**, and their ultimate bin names shown. The areas defined by these marker-containing contigs on the ESOM-map were used to bin additional contigs that did not contain marker genes. Additionally, marker-containing contigs that showed tetranucleotide composition dissimilar to the rest of the cluster were discarded from the bin (for example, see cluster 6). The completely assembled AB1_lowgc chromosome (cluster 9) was included as a control.

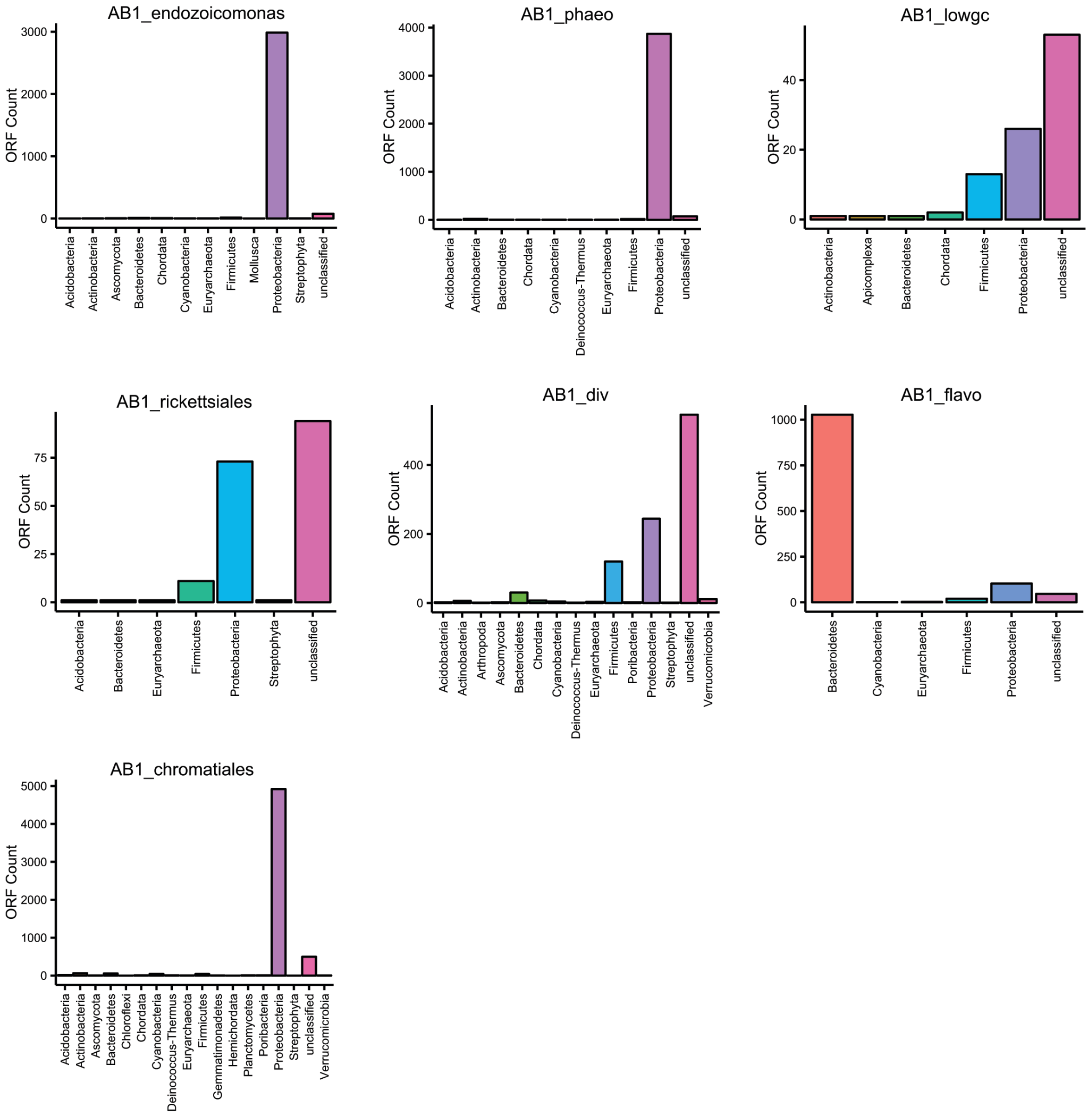

Supplementary Figure 8: Four bacterial genome assembly bins contain ORFs with high taxonomic fidelity, and the other three have low similarity to known sequences and exhibit low fidelity in the taxonomic classification of their ORFs. The histograms above show the phylum-level classification of ORFs in each genome bin. The three divergent genomes (AB1_lowgc, AB1_rickettsiales and AB1_div) are dominated by ORFs unclassified at the phylum level, with others showing inconsistent phylum-level classification.

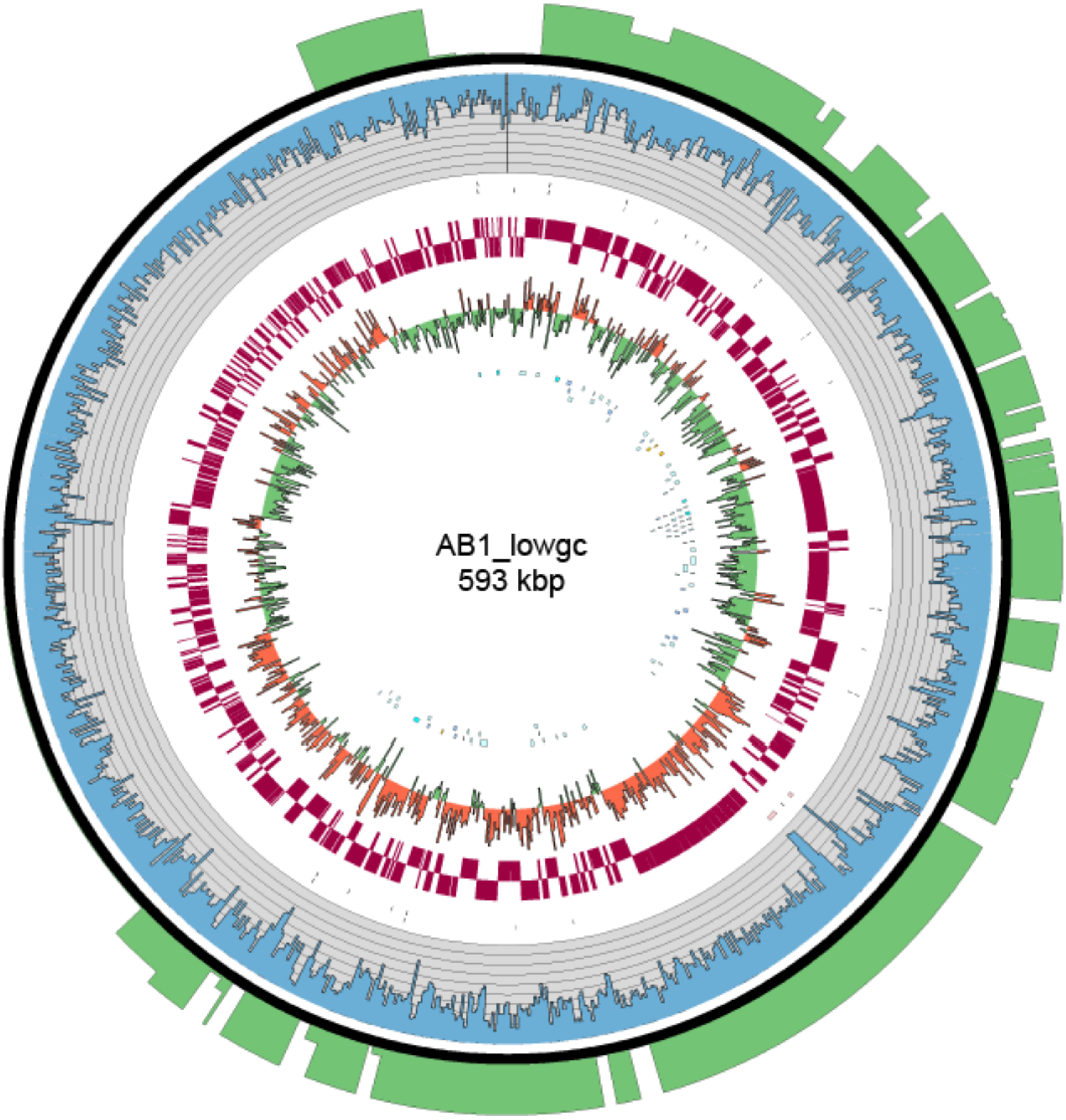

Supplementary Figure 9: Circular map of the AB1_lowgc genome and genome features. Circles correspond to the following (from outermost): (*i*) bar graph showing number of BLAST hits when translated ORFs are queried against the NR database (from 0, inner, to 500, outer). Only 139 out of 610 (22.8%) predicted coding genes had any BLASTP hits with e-values <1× 10 5; (*ii*) GC% using a 500-bp window size (scale from 0, outer, to 100%, inner); (*iii*) tiles showing the location of predicted noncoding RNA genes; (*iv*) red heatmap showing the locations of predicted forward ORFs (outer) and reverse ORFs (inner); (*v*) plot of GC skew (G - C)/(G + C) using a 500-bp window size; (*vi*) tiles showing the location of predicted protein coding genes with assigned functions.

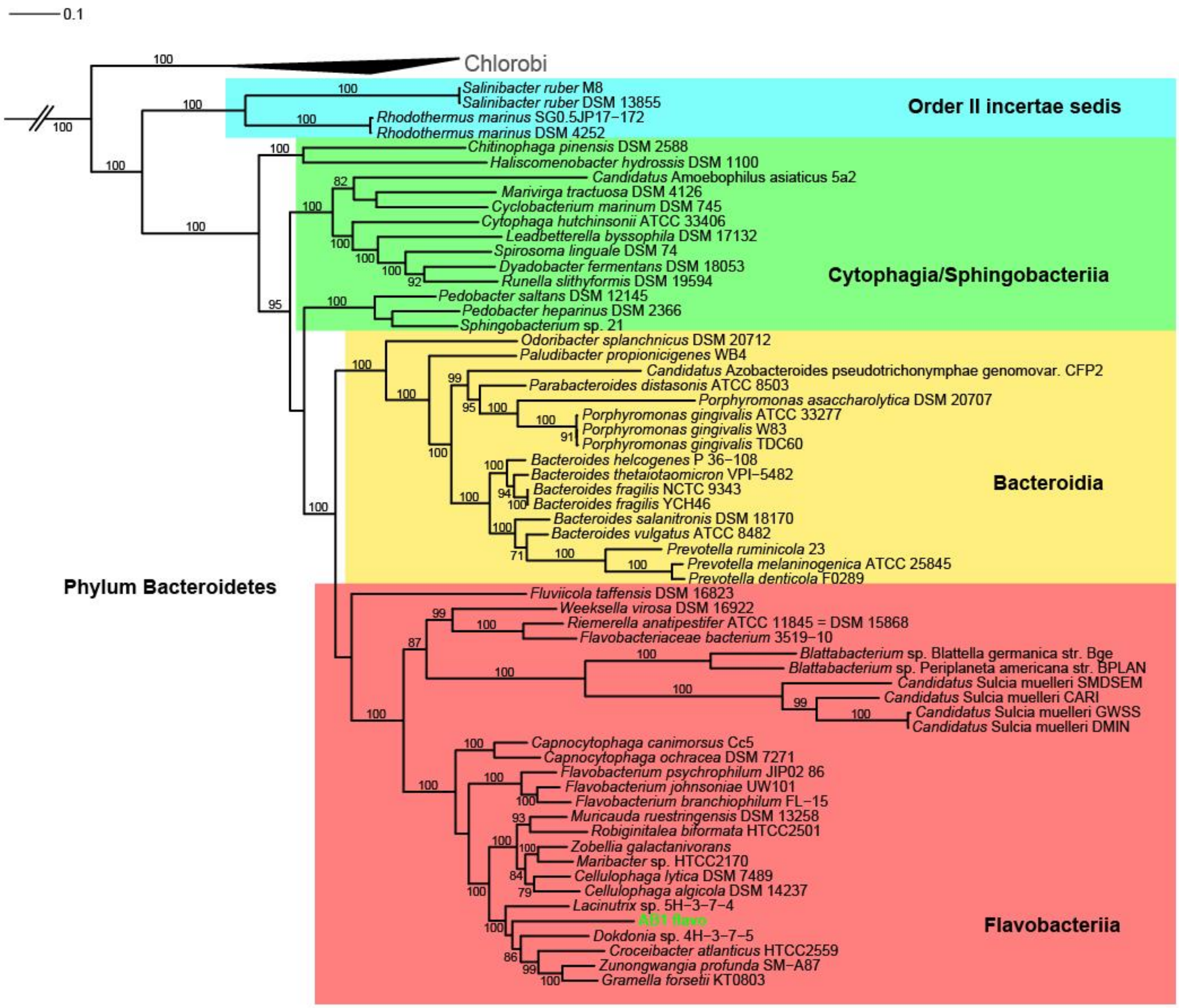

Supplementary Figure 10: An approximately maximum likelihood tree generated by FastTree 2 from concatenated single-copy marker gene protein sequences from the AB1_flavo genome assembly and 1,338 other reference genomes. Bootstrap proportions greater than 70% are expressed to the left of each node as a percentage of 1,000 replicates.

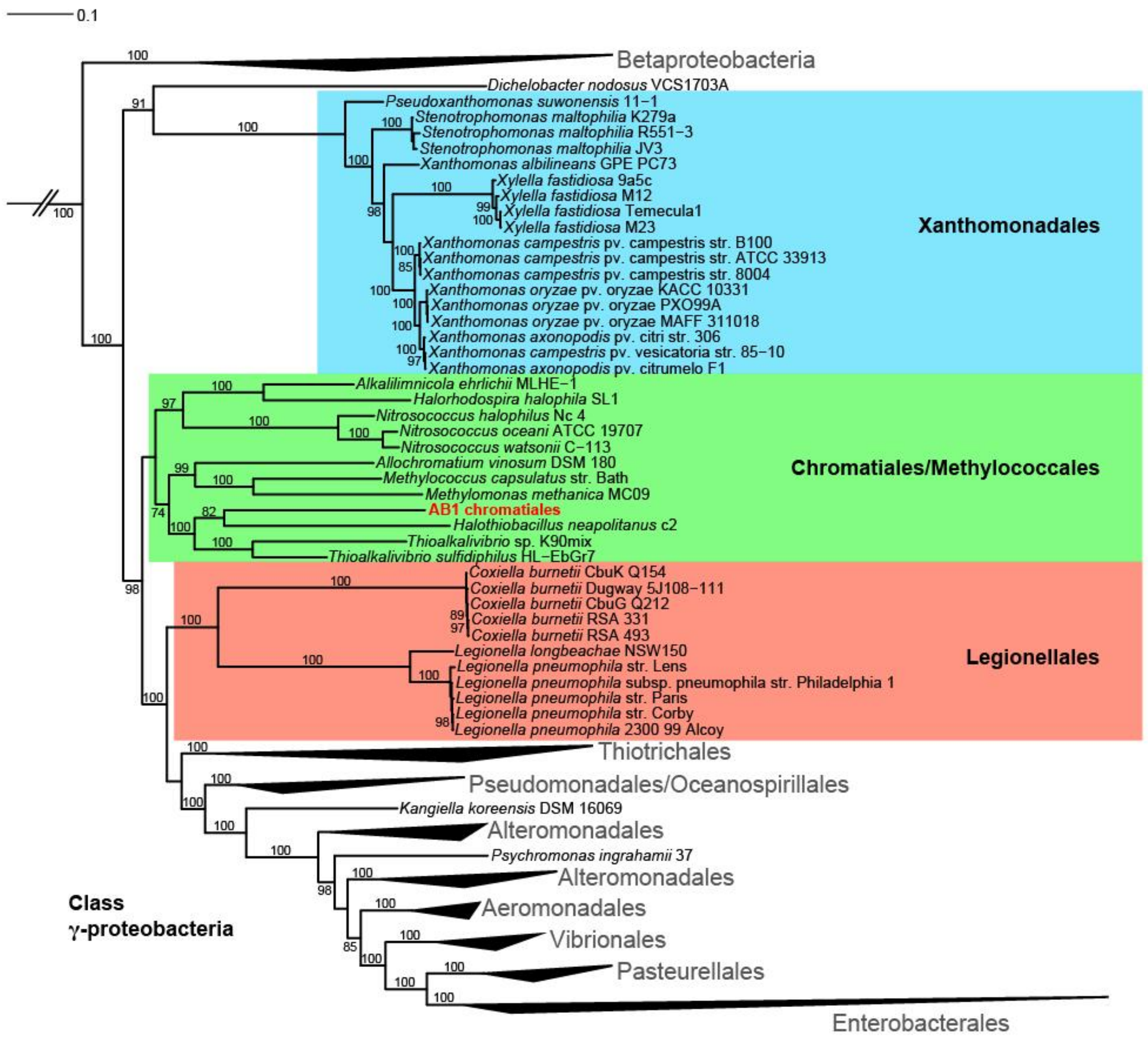

Supplementary Figure 11: An approximately maximum likelihood tree generated by FastTree 2 from concatenated single-copy marker gene protein sequences from the AB1_chromatiales genome assembly and 1,336 other reference genomes. Bootstrap proportions greater than 70% are expressed to the left of each node as a percentage of 1,000 replicates.

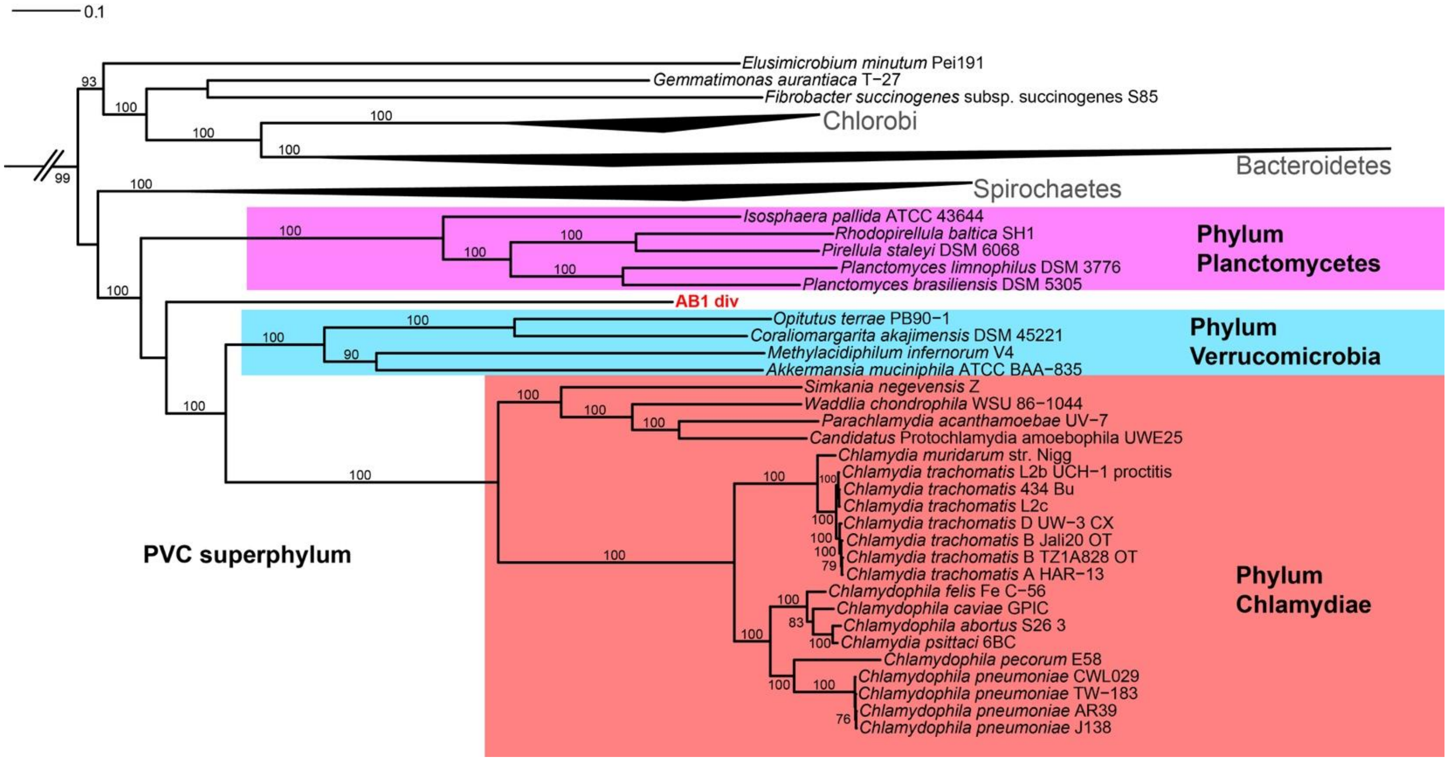

Supplementary Figure 12: An approximately maximum likelihood tree generated by FastTree 2 from concatenated single-copy marker gene protein sequences from the AB1_div genome assembly and 1,336 other reference genomes. Bootstrap proportions greater than 70% are expressed to the left of each node as a percentage of 1,000 replicates.

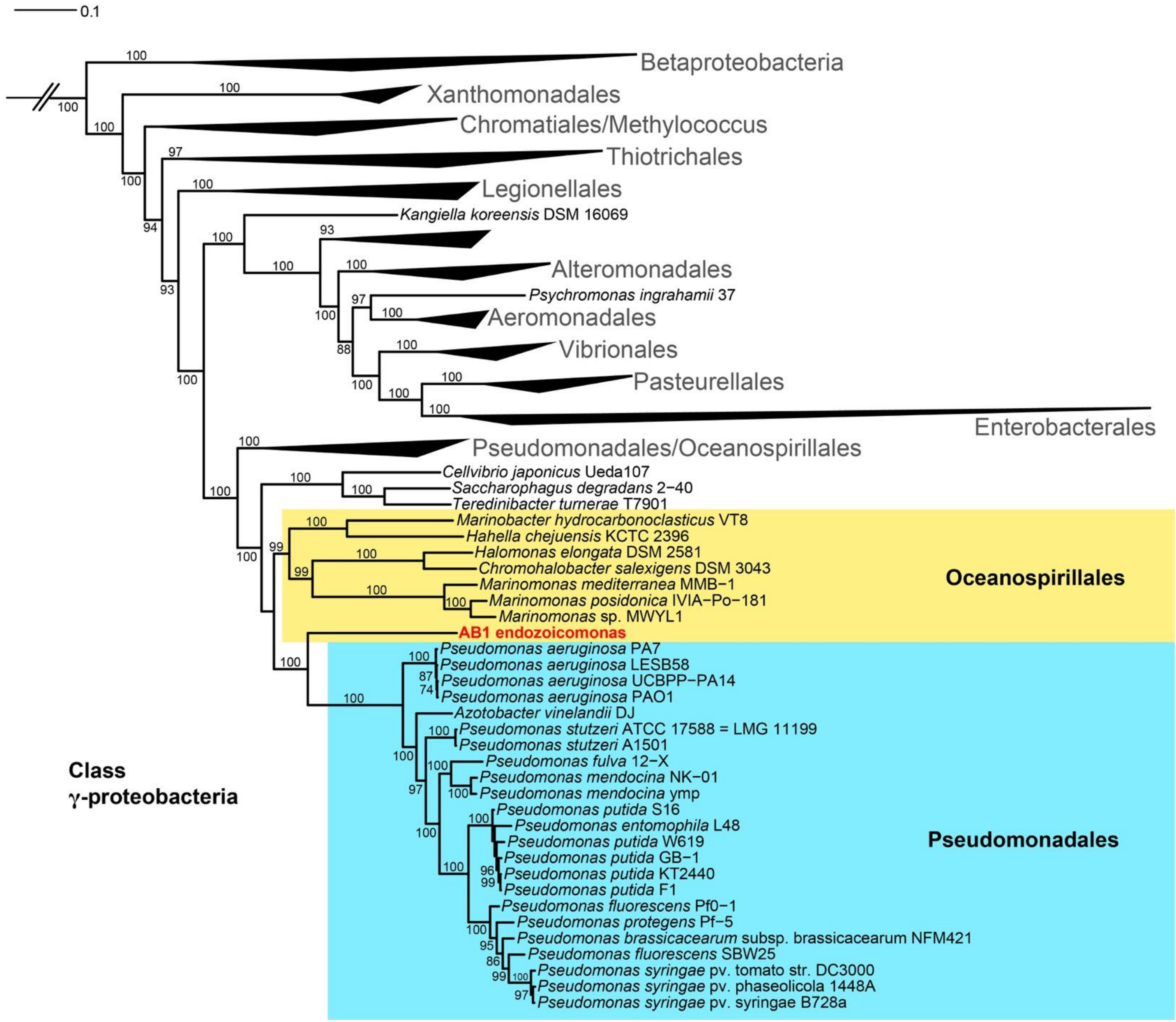

Supplementary Figure 13: An approximately maximum likelihood tree generated by FastTree 2 from concatenated single-copy marker gene protein sequences from the AB1_endozoicomonas genome assembly and 1,333 other reference genomes. Bootstrap proportions greater than 70% are expressed to the left of each node as a percentage of 1,000 replicates.

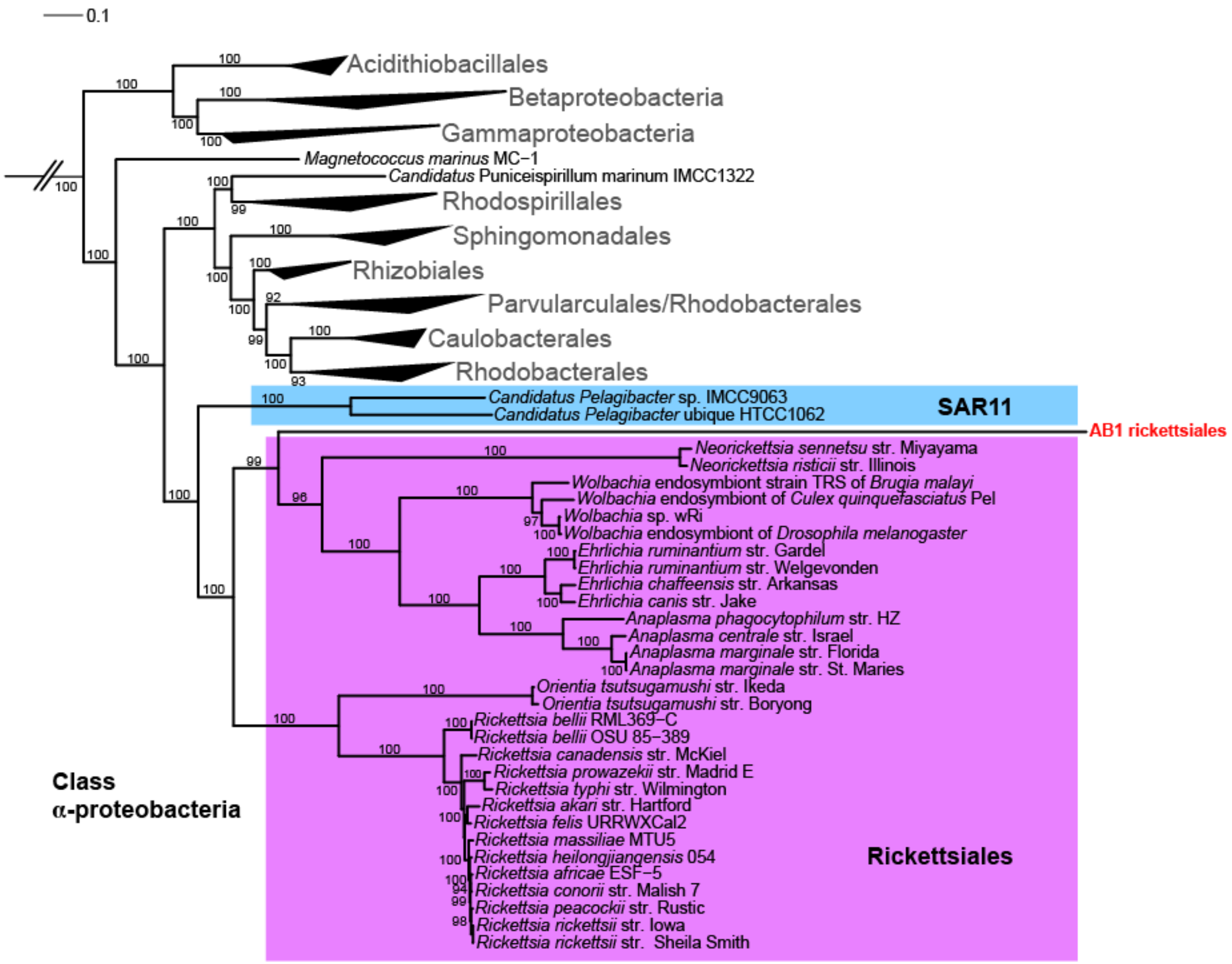

Supplementary Figure 14: An approximately maximum likelihood tree generated by FastTree 2 from concatenated single-copy marker gene protein sequences from the AB1_rickettsiales genome assembly and 1,336 other reference genomes. Bootstrap proportions greater than 70% are expressed to the left of each node as a percentage of 1,000 replicates.

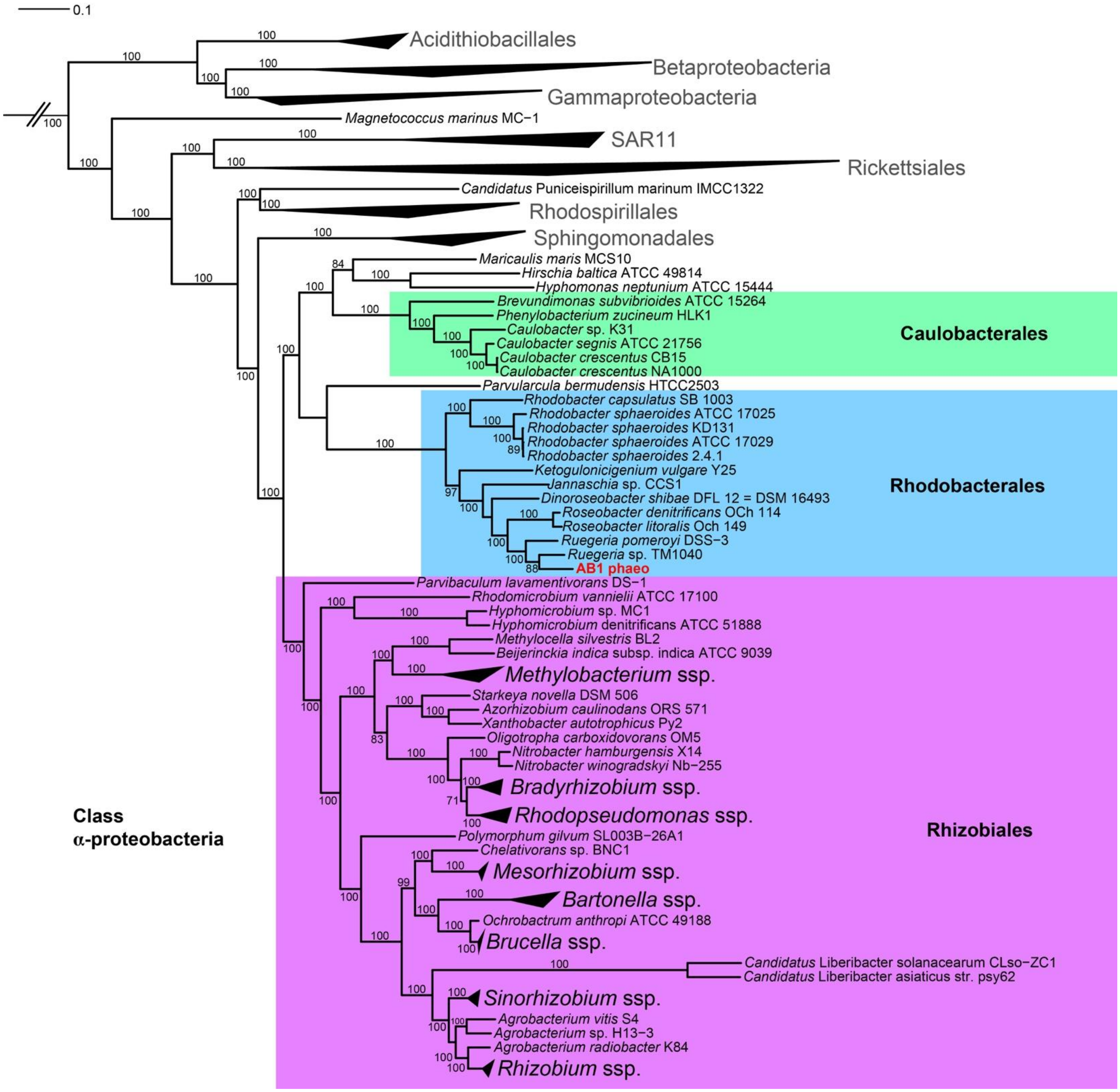

Supplementary Figure 15: An approximately maximum likelihood tree generated by FastTree 2 from concatenated single-copy marker gene protein sequences from the AB1_phaeo genome assembly and 1,336 other reference genomes. Bootstrap proportions greater than 70% are expressed to the left of each node as a percentage of 1,000 replicates.

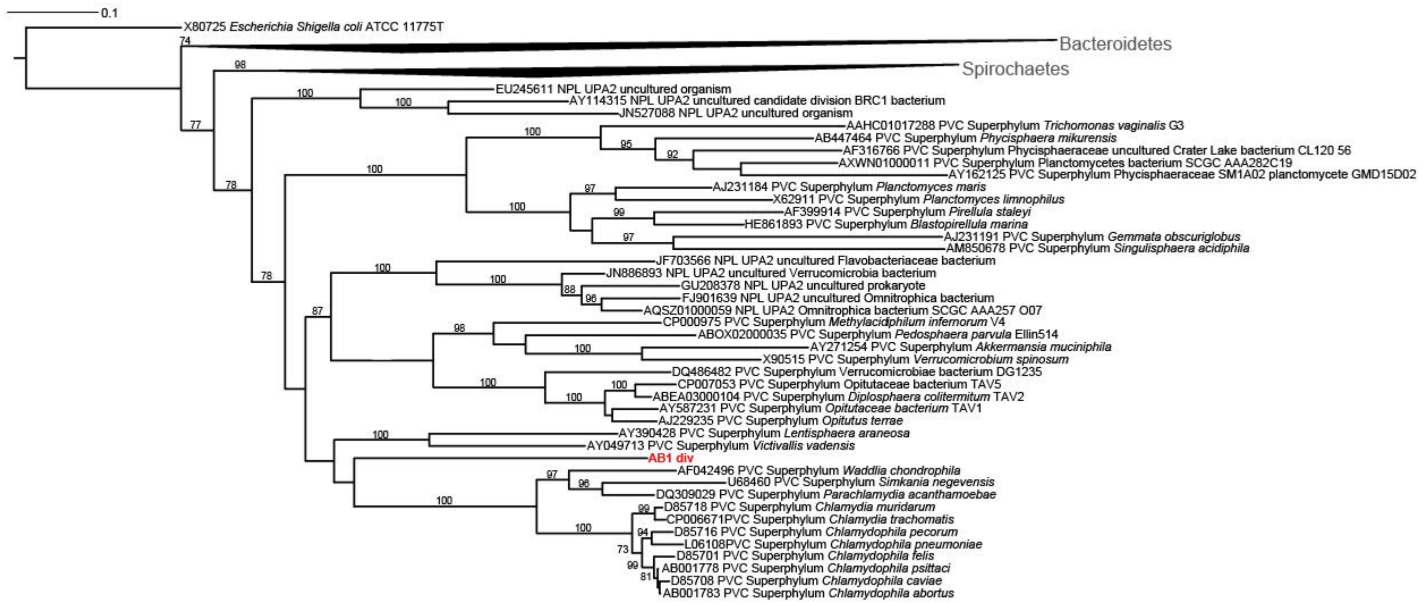

Supplementary Figure 16: An approximately maximum likelihood tree generated by FastTree 2 based on the 16S rRNA gene in the AB1_div genome assembly and 272 other reference sequences. Bootstrap proportions greater than 70% are expressed to the left of each node as a percentage of 1,000 replicates.

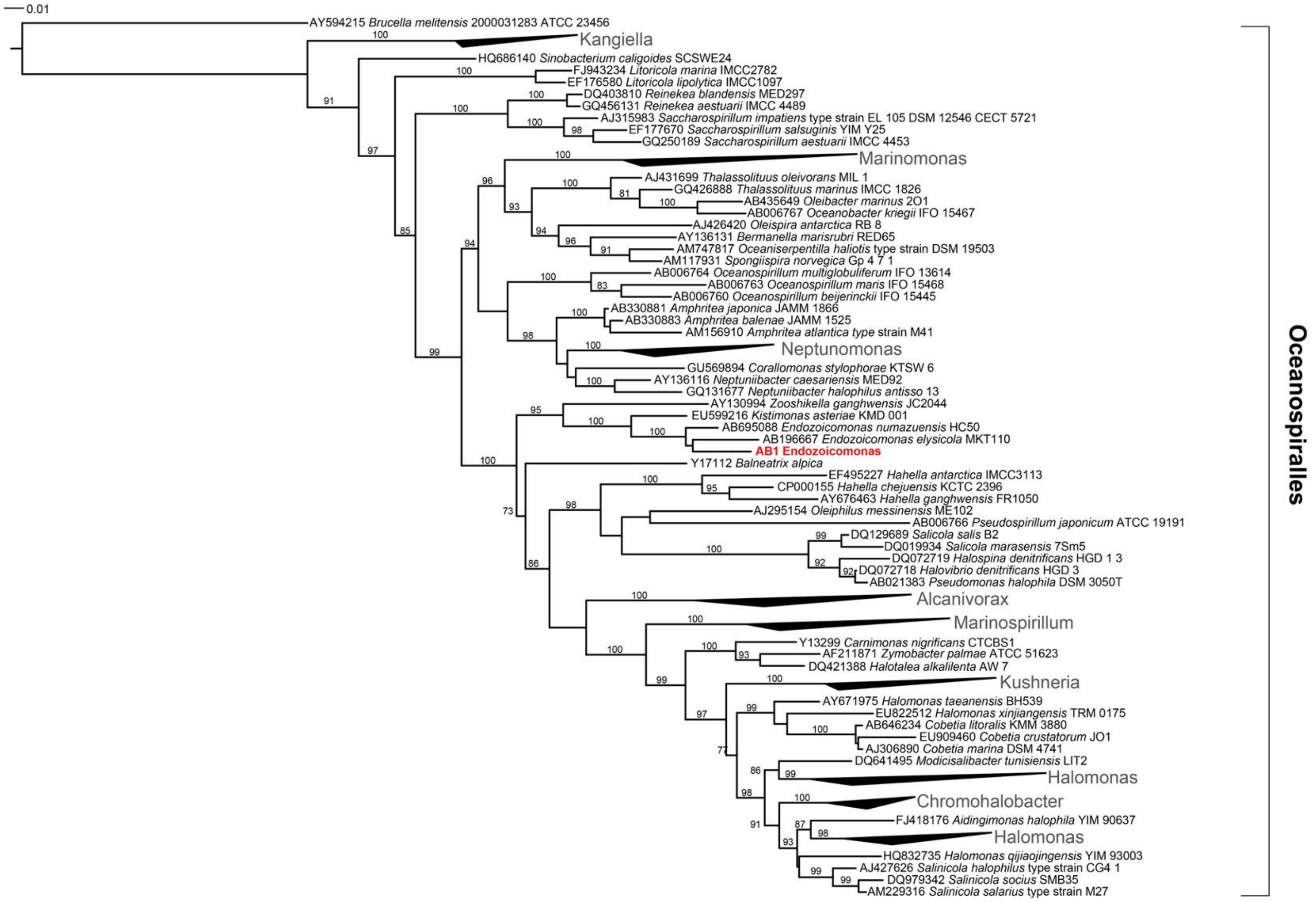

Supplementary Figure 17: An approximately maximum likelihood tree generated by FastTree 2 based on the 16S rRNA gene in the AB1_endozoicomonas genome assembly and 172 other reference sequences. Bootstrap proportions greater than 70% are expressed to the left of each node as a percentage of 1,000 replicates.

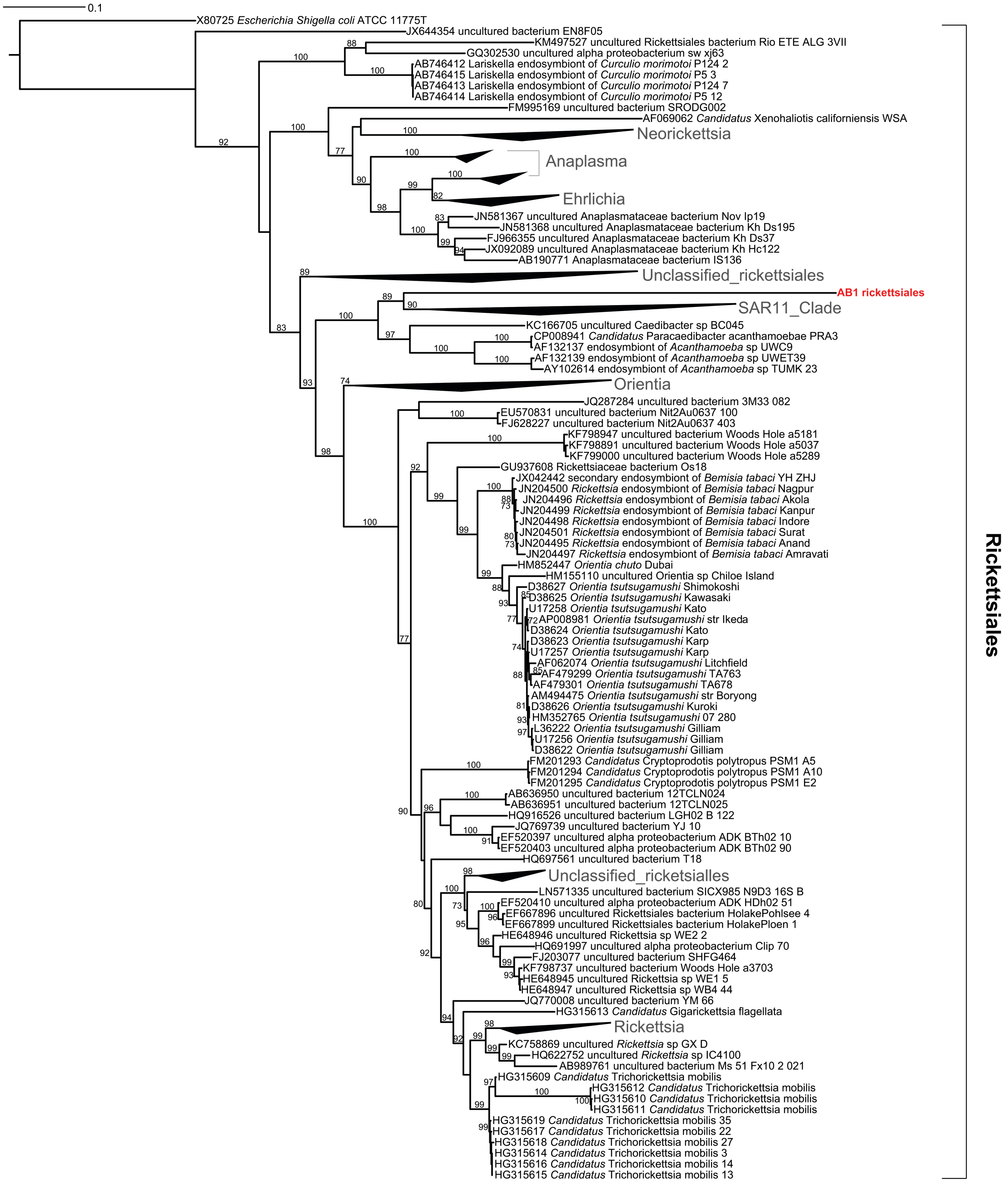

Supplementary Figure 18: An approximately maximum likelihood tree generated by FastTree 2 based on the 16S rRNA gene in the AB1_rickettsiales genome assembly and 691 other reference sequences. Bootstrap proportions greater than 70% are expressed to the left of each node as a percentage of 1,000 replicates.

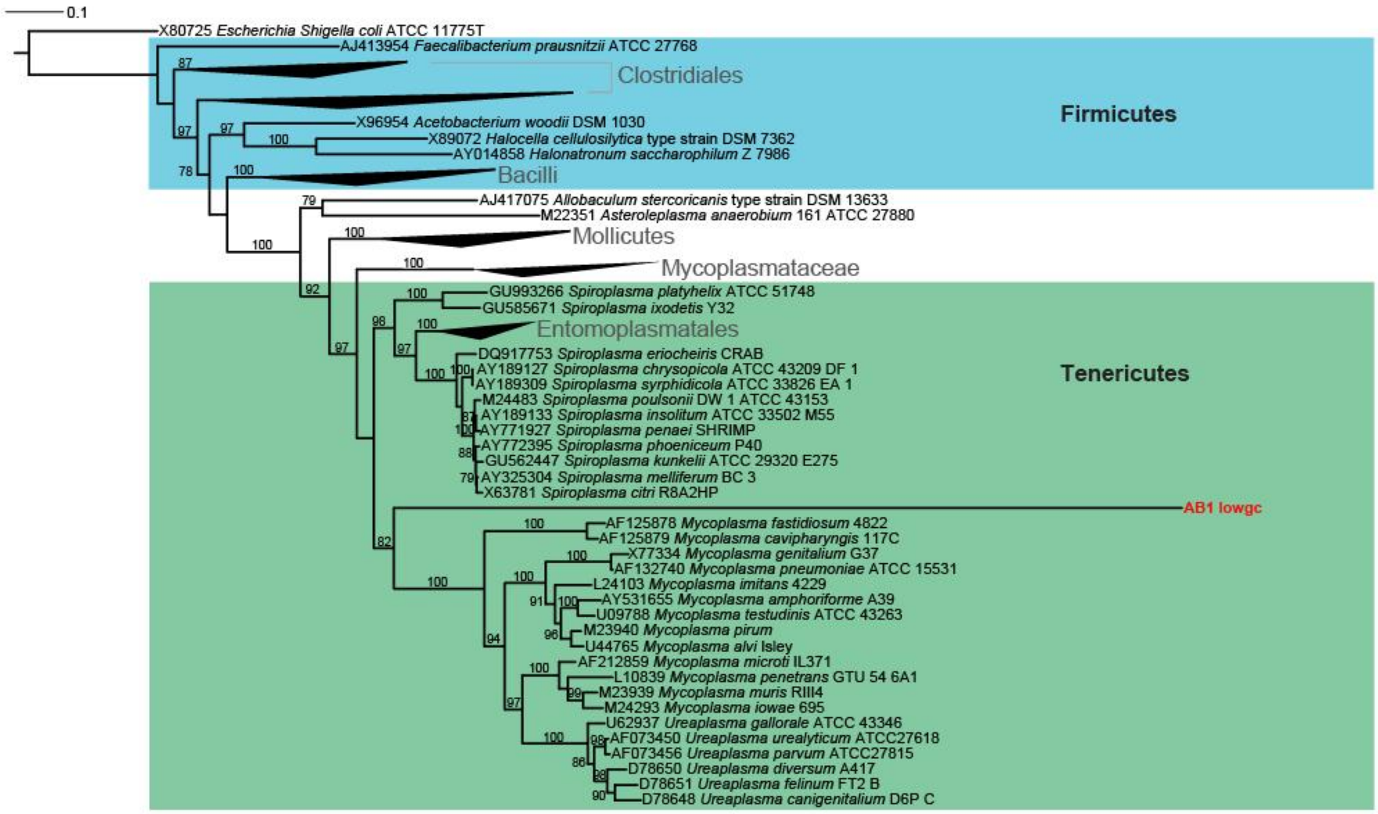

Supplementary Figure 19: An approximately maximum likelihood tree generated by FastTree 2 based on the 16S rRNA gene (without the in the AB1_lowgc genome assembly and 244 other reference sequences. Bootstrap proportions greater than 70% are expressed to the left of each node as a percentage of 1,000 replicates.

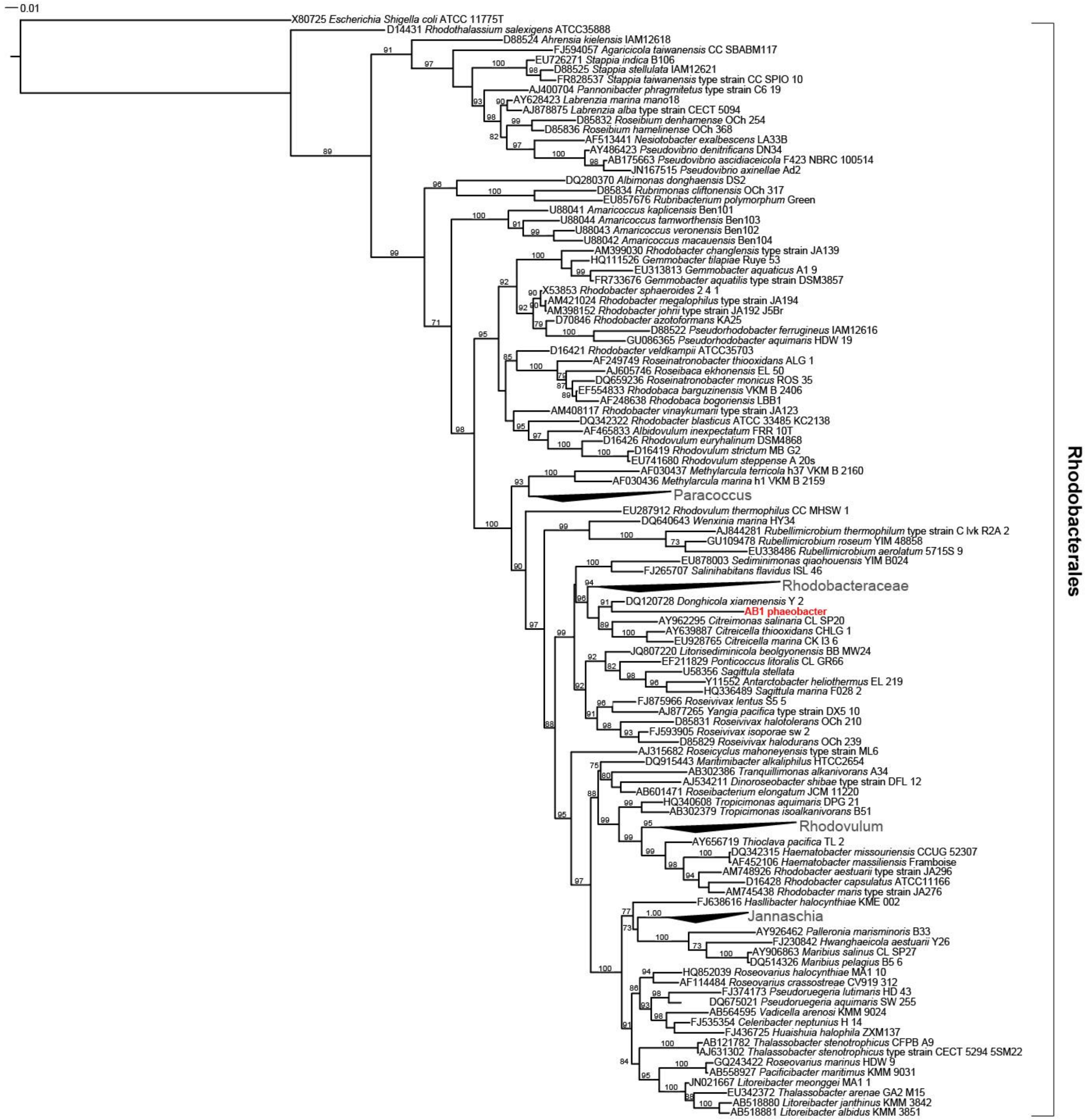

Supplementary Figure 20: An approximately maximum likelihood tree generated by FastTree 2 based on the 16S rRNA gene in the AB1_phaeo genome assembly and 232 other reference sequences. Bootstrap proportions greater than 70% are expressed to the left of each node as a percentage of 1,000 replicates.

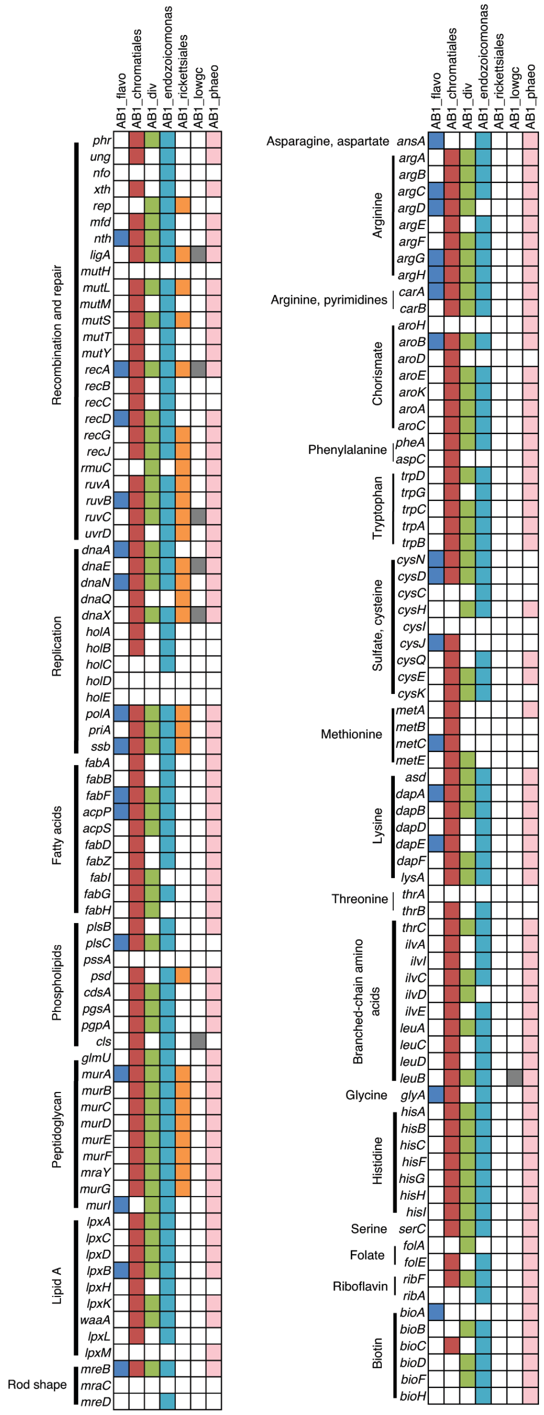

Supplementary Figure 21: Gene inventory analysis. Gene content of assembled genome bins**.** Colored squares show the presence of genes found in annotated genome bins (columns), while white squares indicate an absence of genes (rows).

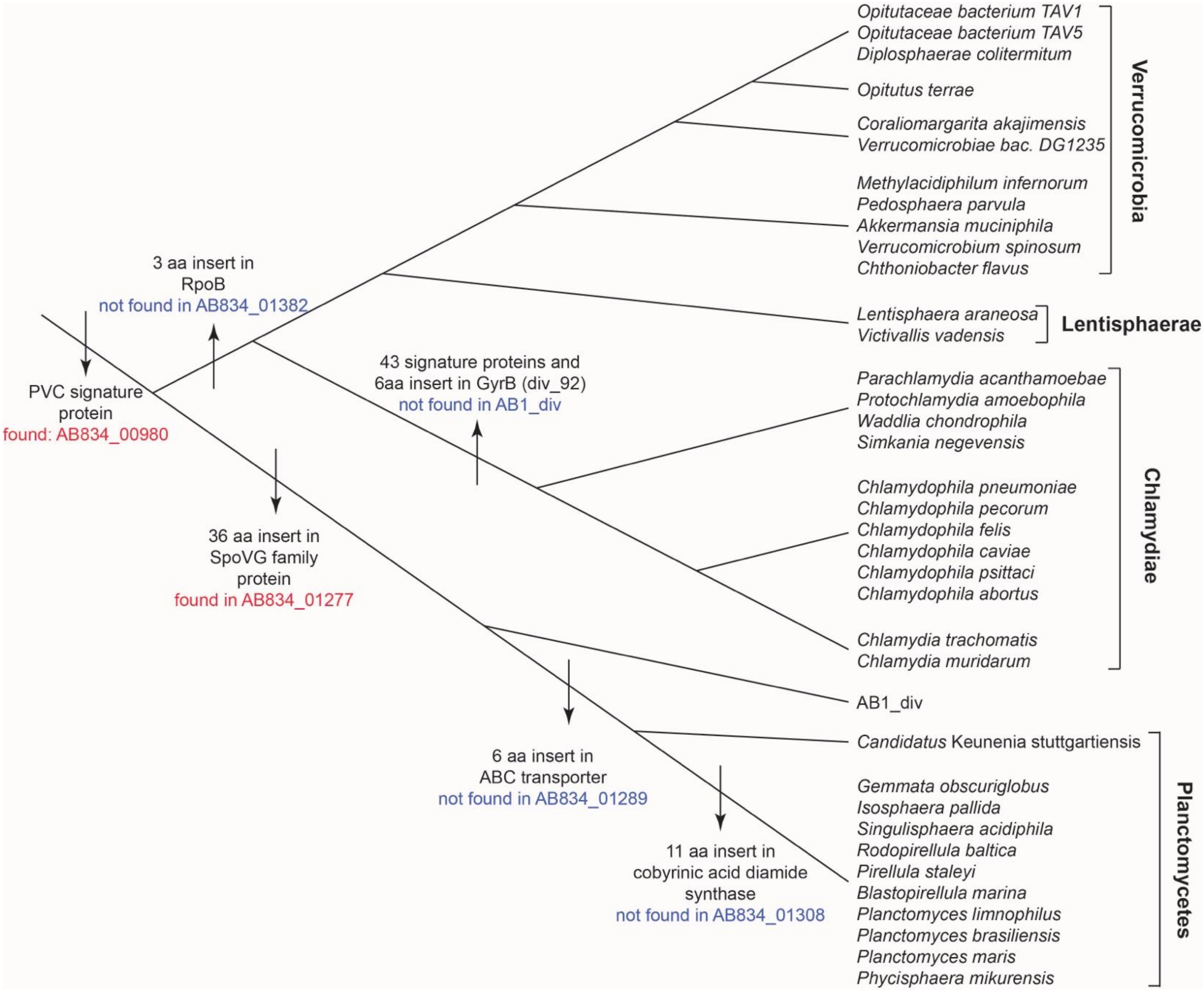

Supplementary Figure 22: Analysis of signature proteins and indels observed in the AB1_div genome places it in the PVC superphylum. A signature protein of the PVC superphylum (29) was found in the AB1_div assembly (AB384_00980), and a 36 amino acid insert was found in a SpoVG family protein, previously found to be specific to the phylum Planctomycetes (30). However, other characteristic Planctomyces inserts - in an ABC transporter, and in cobyrinic acid acdiamide synthase were not found in AB1_div, suggesting that is a basal branch of the Planctomycetes lineage, which is consistent with 16S identity (highest identity is 78% to Planctomyces sequences in the SILVA database). A 3 amino acid insert in RpoB, characteristic of Verrucomicrobia, Lentisphaerae and Chlamydiae (30) was not found in AB1_div, and 43 signature proteins found to be specific to Chlamydiae (31) were also not found in AB1_div.

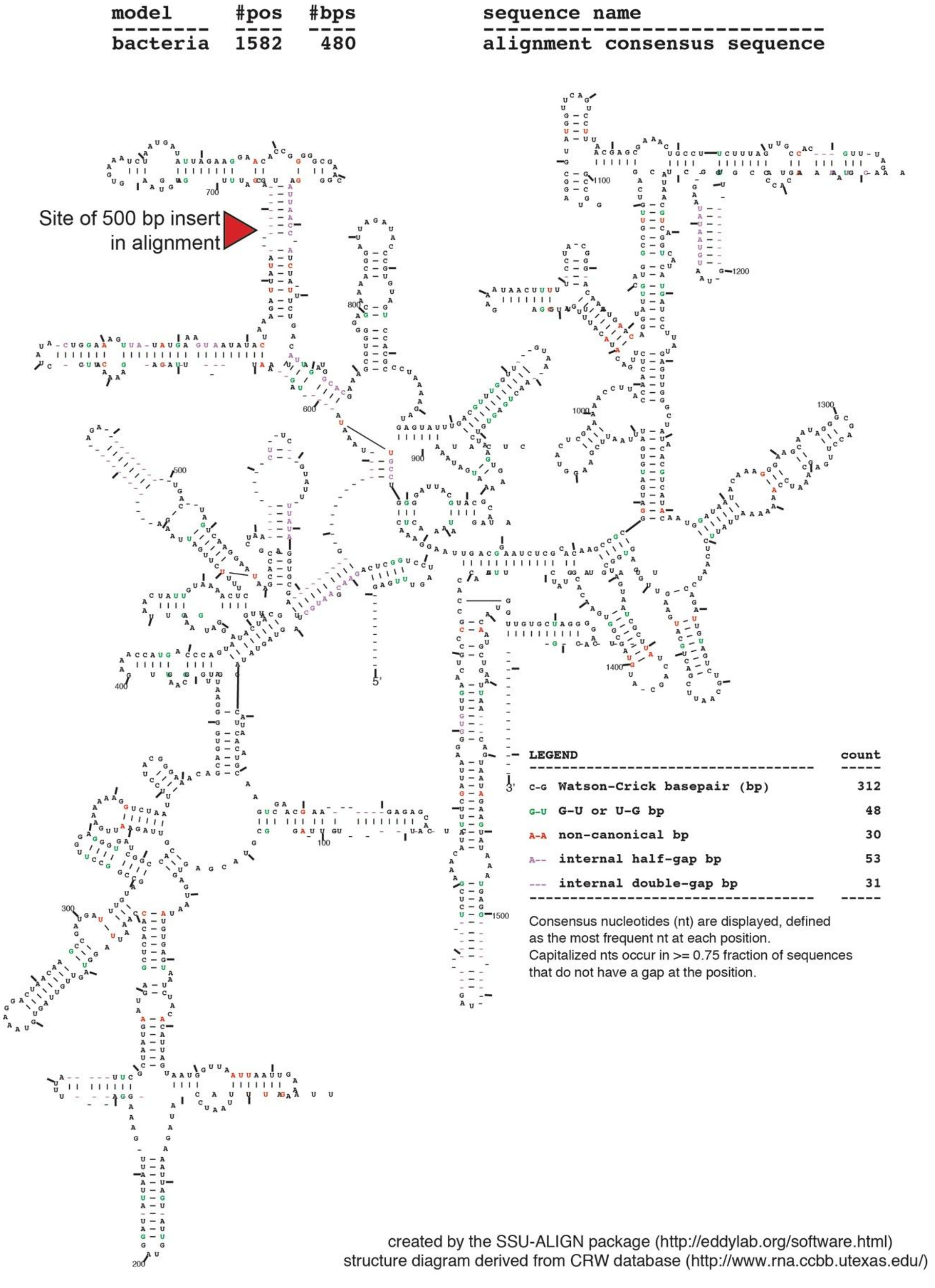

Supplementary Figure 23: Alignment of AB1_lowgc 16S sequence to a structural model of the bacterial small ribosomal subunit, constructed with SSU-Align (19). The location of the 500 bp intervening sequence in the alignment is shown.

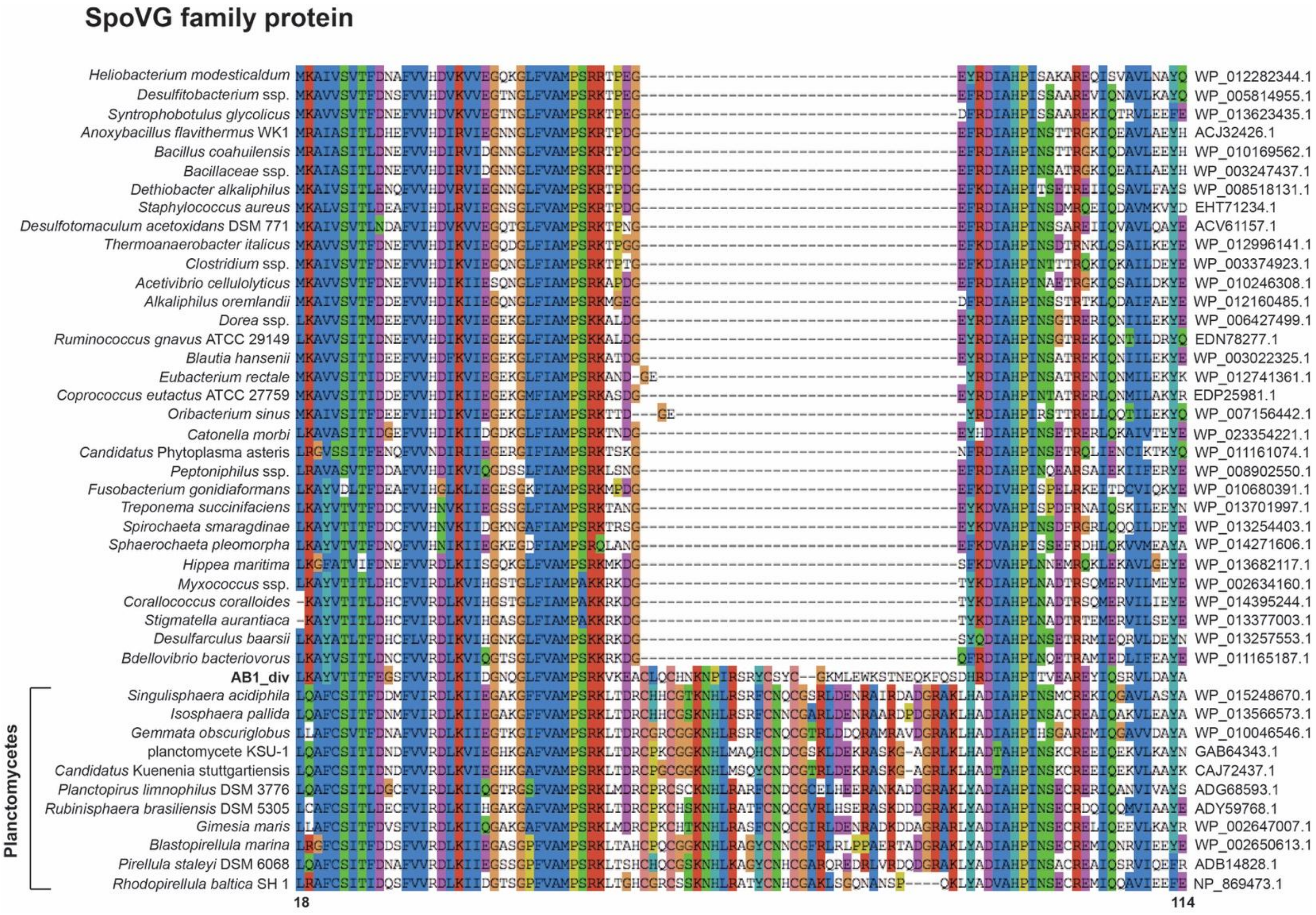

Supplementary Figure 24: Protein alignment of SpoVG family protein, showing region with 36 amino acid insert specific for the Planctomyces branch of the PVC superphylum.

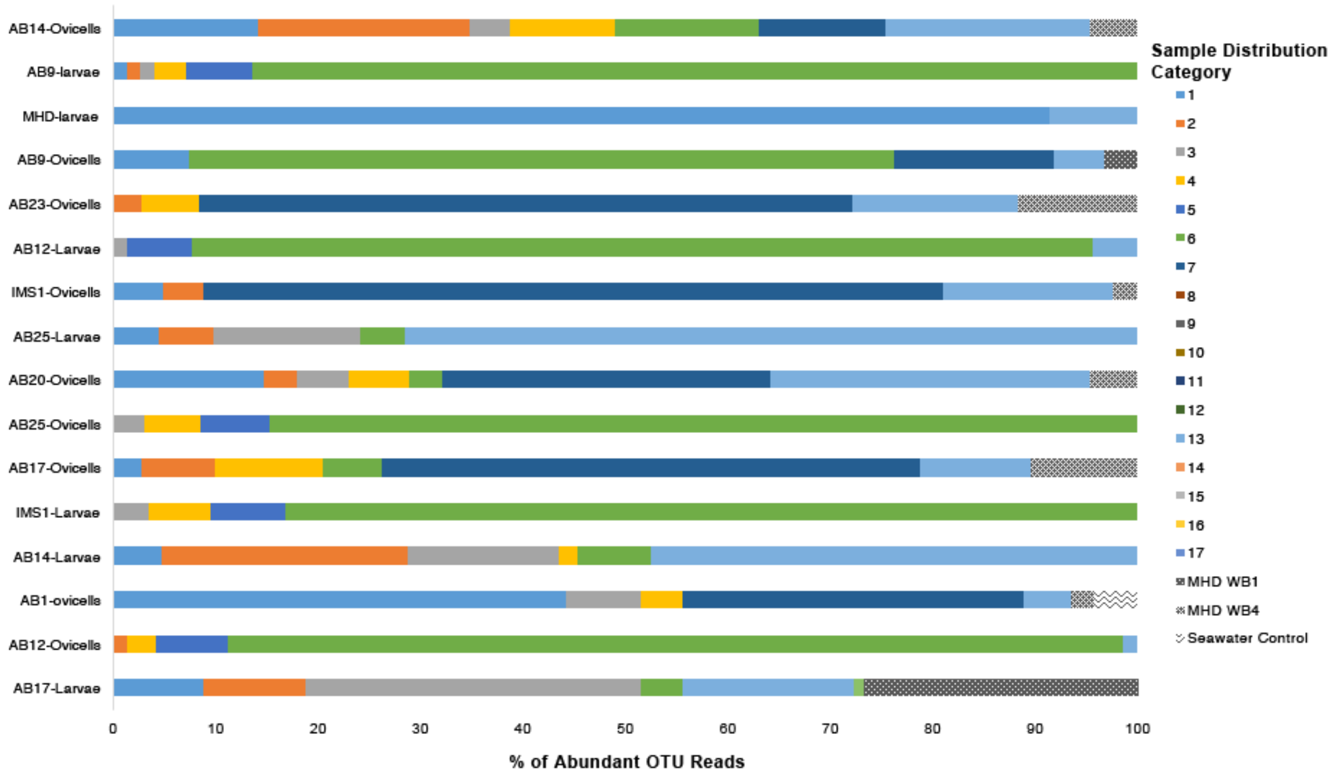

Supplementary Figure 25: Distribution of abundant (> 1%) bacterial OTUs in *Bugula neritina*, *Bugula Stolonifera* and control seawater. The distribution category reflects the number of samples in which a given abundant OTU appears in *B. neritina* samples; if the OTU was also found at abundant levels in *B. stolonifera* (MHD WB1 and MHD WB4) or the seawater control, then it was placed into those respective categories. 44.2% of reads corresponding to abundant bacteria in the AB1_ovicells sample were not detected in any other of the samples subjected to 16S amplicon sequencing.

Supplementary Table 1. Sequence datasets and assembly characteristics

Method of Analysis	AB1_ovicells	MHD_larvae
**16S PCR sequencing**		
Illumina MiSeq reads (2 × 251 bp), thousands	429	382
**Whole metagenome sequencing**		
Illumina HiSeq reads (2 × 101 bp), millions	404.5	219
Illumina HiSeq reads (2 × 101 bp), Gbp	55.8	22.1
Total assembly, thousands of contigs	591.8	505.9
Total assembly, Mbp	498.5	435
Total assembly, N50, bp	2,632	1,638
Contigs >3 kbp, thousands	27.6	19.0
Contigs >3 kbp, Mbp	237.6	172.3
Bacterial contigs >3 kbp	6,372	
Bacterial contigs >3 kbp, Mbp	56.3	
**Whole metatranscriptome sequencing**		
Illumina HiSeq reads (2 × 101 bp), millions	118	
Illumina HiSeq reads (2 × 101 bp), Gbp	11.9	
Illumina HiSeq reads (2 × 151 bp), millions	357	
Illumina HiSeq reads (2 × 151 bp), Gb	53.9	

Supplementary Table 2: Bacterial 16S rRNA sequences reconstructed from the AB1_ovicells metagenomic reads directly by EMIRGE (35) and their assigned genome bin, where they have been joined to assemblies with PCR and Sanger sequencing. Note: The 16S sequences of AB1_rickettsiales and AB1_lowgc that were assembled *de novo* were not reconstructed by EMIRGE, likely because of their divergence from sequences in the SILVA database (26).

Sequence	***Relative Abundance***	***Best BLASTN hit (accession, identity)***	***RDP classification (confidence)***	***Assigned genome bin***
41	0.45	Uncultured bacterium clone BA100-C1-seq (JX280191.1, 97%)	Genus: *Endozoicomonas* (100%)	AB1_endozoicomonas
4019	0.12	Uncultured Rhodobacteraceae bacterium clone MD2.45 (FJ403094.1, 96%)	Genus: *Roseovarius* (99%)	
96	0.10	Endobugula sertula strain BnSP (AF006606.2, 99%)	Genus: *Eionea* (99%)	AB1_endobugula
199	0.089	Uncultured bacterium clone SanDiego a7349 (KF799885.1, 92%)	Genus: *Phaeobacter* (41%)	AB1_phaeo
128	0.062	Uncultured gamma proteobacterium clone 27D24 (GQ274161.1, 96%)	Genus: *Bermanella* (33%)	
145	0.058	Uncultured bacterium clone 5S1 (JF272174.1, 96%)	Genus: *Granulosicoccus* (100%)	
281	0.041	Uncultured organism clone ctg CGOFF0066 (DQ395743.1, 91%)	Genus: *Parachlamydia* (27%)	AB1_div
164	0.041	Uncultured Sphingobacteriales bacterium clone B255 A11 (EF092220.1, 96%)	Genus: *Nitritalea* (11%)	
233	0.033	*Amphritea* sp. MEBiC05461T 16S (GU289646.1, 98%)	Genus: *Amphritea* (100%)	

Supplementary Table 3: Automated MaxBin (41) binning results for AB1_ovicells bacterial contigs >3 kbp, displaying a high number of repeated markers compared to binning based on normal mixture modeling (11) (**Main Paper, Table 1**).

Cluster Name/Number	***Relative Abundance***	***Completeness (%)***	***Genome size (bp)***	***GC content (%)***	***No. Repeated markers***	***No. contigs***
AB1_bacteria_over3k.001.fasta	23.89	94.40%	4164677	35	8	313
AB1_bacteria_over3k.002.fasta	17.24	44.90%	1007589	21	16	29
AB1_bacteria_over3k.003.fasta	6.04	100.00%	4869462	45	40	399
AB1_bacteria_over3k.004.fasta	4.12	94.40%	4750895	42	45	483
AB1_bacteria_over3k.005.fasta	2.93	68.20%	3707167	41	18	514
AB1_bacteria_over3k.006.fasta	2.7	96.30%	5119588	60	13	321
AB1_bacteria_over3k.007.fasta	1.97	38.30%	2772797	34	6	435
AB1_bacteria_over3k.008.fasta	1.78	13.10%	1725527	39	1	237
AB1_bacteria_over3k.009.fasta	1.61	17.80%	2086909	43	6	452
AB1_bacteria_over3k.010.fasta	1.41	57.90%	6393031	51	30	1309

Supplementary Table 4: Primers used in this study

Name	***Sequence***	***Citation***	***Notes***
S-D-Bact-0341-b-S-17	CCTACGGGNGGCWGCAG	Klindworth *et al. (38)*	16S rRNA amplicon sequencing
S-D-Bact-0785-a-A-21	GACTACHVGGGTATCTAATCC	Klindworth *et al. (38)*	16S rRNA amplicon sequencing
AB1_div_F	CTAGTTATGTAGTTCTGG	This study	Detection of AB1_div
AB1_div_R	GGCTTTCGAGTCGTAAAAC	This study	Detection of AB1_div
AB1_lowGC_16S_576F	GCTTGTGCGAGATTCCGT	This study	Detection of AB1_lowgc
AB1_lowGC_16S_730R	ACGGATTAGATACCCGTG	This study	Detection of AB1_lowgc
Bn240f	TGCTATTTGATGAGCCCGCGTT	Haygood & Davidson (40)	Detection of *Ca.* E. sertula (AB1_endobugula)
Bn1253r	CATCGCTGCTTCGCAACCC	Haygood & Davidson (40)	Detection of *Ca.* E. sertula (AB1_endobugula)
EndozoiF	TGCGTAGGCGGCTCGTTAAGTT	This study	Detection of AB1_endozoicomonas; Detecting and sanger sequencing of connectivity between 16S and contig
EndozoiR	AATTCGCAGGATGTCAAGGCC	This study	Detection of AB1_endozoicomonas
Low1_L2	AGGTTTAGCAGAATAAGTTGGA	This study	Closing circular AB1_lowgc chromosome
Low1_64_ R2	GGTTTTATAAGCCCTGACCA	This study	Closing circular AB1_lowgc chromosome
AB1_phaeo_16S_302F	CTCTTTCGCCTGTGATGATA	This study	Detecting connectivity between AB1_phaeo 16S and contig
Phaeo_ribo_546_R	CCAAGAAAAATCCATGTCCG	This study	Detecting connectivity between AB1_phaeo 16S and contig

AB1_endoz_5S_105R	CCTACTCTCACATGGGGATA	This study	Detecting connectivity between AB1_endozoicomonas 16S to contig
AB1_lowcov_5S_608R*	ATCGCTTTTACTGCCTAGTT	This study	Detecting connectivity between AB1_rickettsiales 16S and contig
AB1_lowcov_235F*	GATTGTAGCTGGTCTGAGAG	This study	Detecting connectivity between AB1_rickettsiales 16S and contig

***Note:** We initially referred to the AB1_rickettsiales genome as "AB1_lowcov," and so these primer names are derived from the original bin name.

Supplementary Table 5: Presence of universal bacterial 16S primer and probe binding sites in genome bins. Dashes denote situations where the EMIRGE (35) reconstructed 16S rRNA sequence does not extend to the primer binding site.

Bin	***27F*** *(36)*	***1492R*** *(37)*	***S-D-Bact-0341-b-2-17*** *(38)*	***S-D-Bact-0785-a-A-21****(38)*	***EUB338*** *(39)*
AB1_div	YES	NO	YES	YES	NO
AB1_endozoicomonas	-	-	YES	YES	YES
AB1_endobugula	-	-	YES	YES	YES
AB1_rickettsiales	YES	NO	YES	YES	NO
AB1_lowgc	YES	NO	NO	YES	NO
AB1_phaeo	-	-	YES	YES	YES

Supplementary Table 6: Genome bin characteristics, prior to iterative assembly

Bin	***No. Contigs***	***Size (Mbp)***	***N50 (kpb)***	***Longest Contig (kpb)***	***Coverage****	***GC%***	***Completeness (%)***	***No. Duplicate markers***
AB1_chromatiales	352	7.29	34.0	209.5	2.4×	50.4	98.6	2
AB1_rickettsiales	25	0.400	20.5	61.7	2.3×	21.4	48.9	0
AB1_phaeo	195	4.55	48.9	257	2.9×	60.4	99.3	3

* The coverage quoted here is k-mer coverage reported by the SPAdes assembler, where k = 77.

Supplementary Table 7: Analysis of genetic code in AB1_lowgc

	Code 4 ***Average ORF length (bp)***	***Average ORF cscore***	***Code 11*** ***Average ORF length (bp)***	***Average ORF cscore***
AB1_lowgc	850	139.1	847	143.2
Ca. Phytoplasma australiense	794	108.8	786	116.5
Mycoplasma pneumoniae	975	117.1	458	55.9

Supplementary Table 8: Genome annotation characteristics

Bin	***No. CDS***	***No. hypothetical genes***	***Average CDS size (bp)***	***No. rRNA***	***No. tRNA***	***Coding Density (%)***
AB1_endozoicomonas	3,525	1,490	962	2	41	83.8
AB1_phaeo	4,425	1,882	921	1	40	87.2
AB1_lowgc	610	481	840	2	30	86.4
AB1_rickettsiales	409	186	951	2	38	89.3
AB1_div	1,341	531	1,119	3	88	78.7
AB1_flavo	1,522	1,052	751	0	7	71.7
AB1_chromatiales	6,560	2,797	977	0	39	86.2
